# Inflation of tumor mutation burden by tumor-only sequencing in under-represented groups

**DOI:** 10.1038/s41698-021-00164-5

**Published:** 2021-03-19

**Authors:** Yan W. Asmann, Kaushal Parikh, P. Leif Bergsagel, Haidong Dong, Alex A. Adjei, Mitesh J. Borad, Aaron S. Mansfield

**Affiliations:** 1 Division of Biomedical Statistics and Informatics, Department of Health Sciences Research, Mayo Clinic, Jacksonville, FL USA; 2 Precision Cancer Therapeutics of Mayo Clinic’s Center for Individualized Medicine, Rochester, MN USA; 3 John Theurer Cancer Center, Hackensack University Medical Center, Hackensack, NJ USA; 4 Division of Hematology and Medical Oncology, Department of Medicine, Mayo Clinic, Scottsdale, AZ USA; 5 Departments of Immunology and Urology, Mayo Clinic, Rochester, MN USA; 6 Division of Medical Oncology, Department of Oncology, Mayo Clinic, Rochester, MN USA

**Keywords:** Diagnostic markers, Cancer genetics

## Abstract

With the recent FDA approval of tumor mutational burden-high (TMB-H) status as a biomarker for treatment with a PD-1 inhibitor regardless of tumor type, accurate assessment of patient-specific TMB is more critical now more than ever. Using paired tumor and germline exome sequencing data from 701 patients newly diagnosed with multiple myeloma, including 575 self-reported White patients and 126 self-reported Black patients, we observed that compared to the gold standard of filtering germline variants with patient-paired germline sequencing data, TMB estimates were significantly higher in both Black and White patients when using public databases for filtering non-somatic mutations; however, TMB was more significantly inflated in Black patients compared to White patients. TMB as a biomarker for patient selection to receive immune checkpoint inhibitors (ICIs) therapy without patient-paired germline sequencing may introduce racial bias due to the under-representation of minority groups in public databases.

Immune checkpoint inhibitors (ICIs) have dramatically improved the survival of patients with many types of cancer. Since the auto-immune toxicities with ICIs can be fatal, it is critical to optimize patient selection criteria. The current use of PD-L1 expression levels and mismatch-repair/microsatellite-instability status has limitations. Response to ICIs is predicated upon mutations that are translated into neoantigens that are presented by tumor cells and recognized by T cells that can eliminate tumor cells. Defective DNA repair leads to higher tumor mutational burden (TMB) which is defined as the total number of nonsynonymous mutations per megabase (Mb) of coding regions of a tumor genome, and is a surrogate for cancer neoantigens that can be recognized by the adaptive immune system. TMB was reported to predict survival after immunotherapy across multiple cancer types^1^. In June 2020, the United States Food and Drug Administration (FDA) approved the use of TMB-high (TMB-H) status as a patient selection criterion for treating adult and pediatric patients with unresectable or metastatic tumors with the PD-1 inhibitor pembrolizumab based on results from the phase 2 KEYNOTE-158 study^2^. Therefore, it is more critical now than ever to have accurate assessment of patient specific TMB.

Currently there is no globally accepted, standardized approach for TMB calculation. The most accurate TMB estimate requires patient-paired germline sequencing to filter out non-somatic variants^3^. However, since patient germline DNAs (e.g. peripheral blood) are not routinely collected in clinic for germline analysis, TMB is often calculated from tumor-only sequencing relying on public germline variant databases (DBs) to filter out non-somatic polymorphisms. We previously reported that filtering based on public DBs significantly inflated TMB^4^. In this study, we investigated the impact of minority group representation in these DBs and hypothesized that TMB would be more greatly inflated in under-represented groups.

The lack of representation of diverse ancestral backgrounds in genomic research, including individuals of African ancestry, is well known^5,6^. Of more than 60,000 individuals genotyped and sequenced, only 8.6% are of African ancestry while 54.9% are of non-Finnish European ancestry.

## Comparative estimations of TMB

**Fig. 1:**
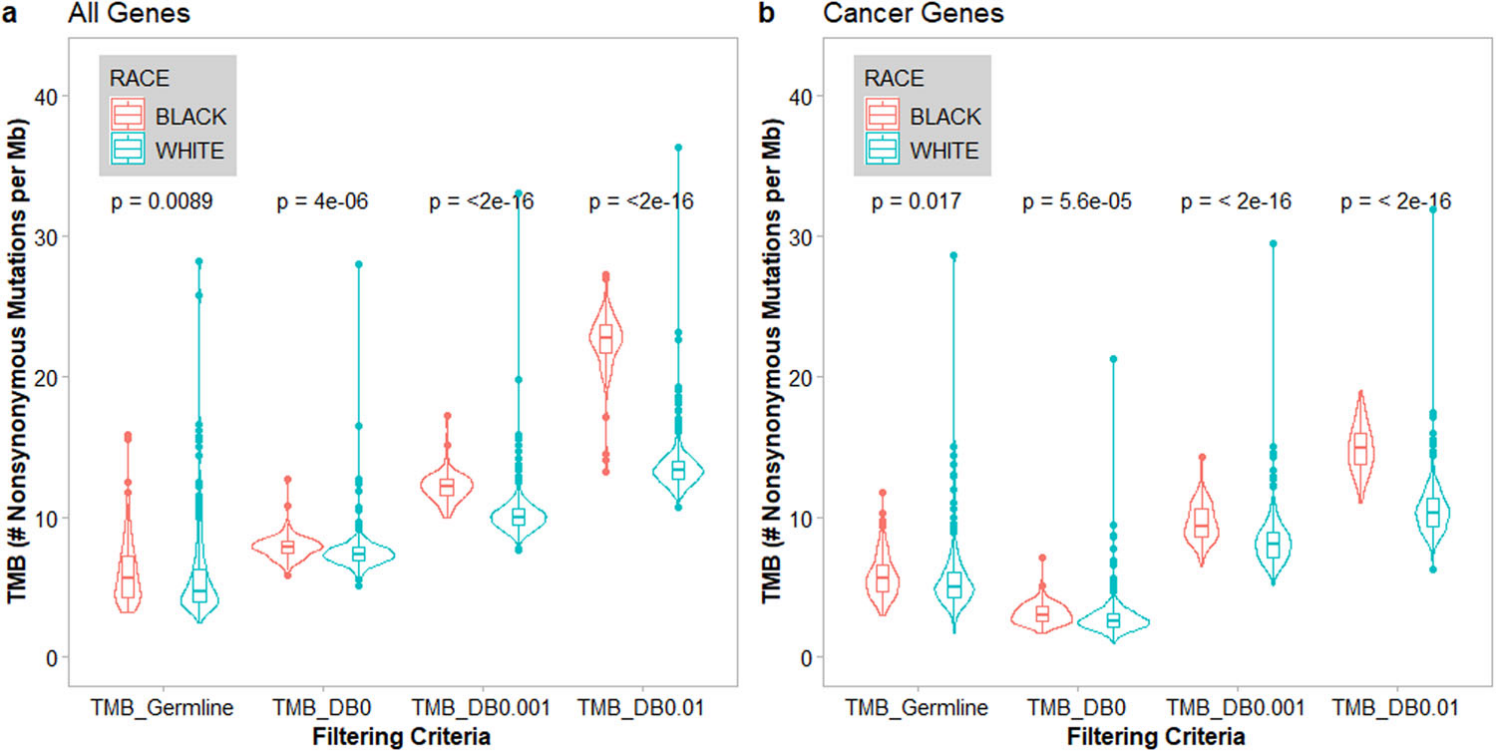
Impact of variant filtering criteria on TMB calculation. The TMB values were calculated as number of nonsynonymous mutations per Mb of coding regions. Four criteria were applied to identify patient-specific somatic mutations: (1) TMB_Germline: excluding variants in patient-matched germline exome; (2) TMB_DB0: excluding all variants reported by 1000G or ExAC; (3) TMB_DB0.001: excluding variants with MAF ≥ 0.1% in 1000G or ExAC; and (4) TMB_DB0.01: excluding variants with minor allele frequency (MAF) ≥ 1% in 1000G or ExAC DBs. The violin and box plots in red are TMB values from Black patients, and blue are from White Patients. **a** Comparisons of TMBs calculated from all protein-coding genes in Black and White patients from four variant filtering criteria. **b** Comparisons of TMB calculated from 1059 cancer genes in Black and White patients from four variant filtering criteria.

**Fig. 2:**
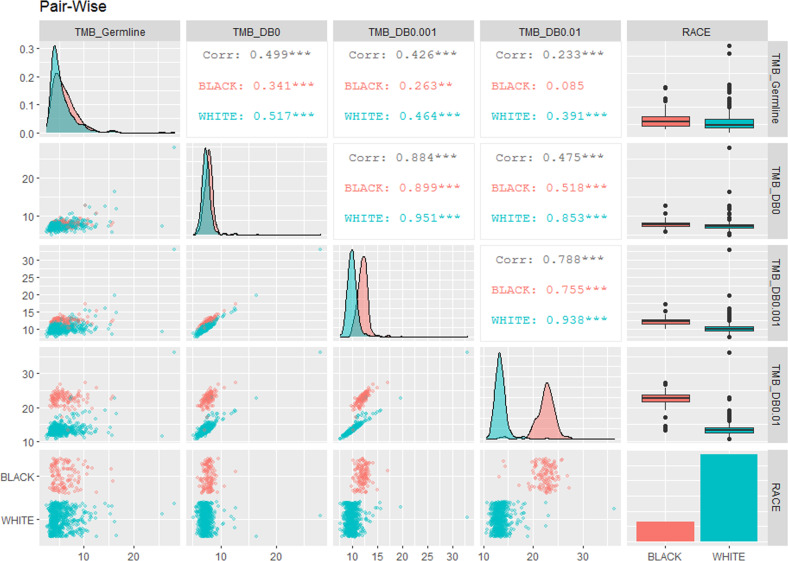
Pair-wise visualization of the differences in tumor mutational burdens between race and variant filtering criteria. This is a 5 × 5 matrix of pair-wise comparison. Red colors are data from Black patients, and blue colors are data from White patients. (1) The diagonal: density plots showing the increased separation of TMB distributions while relaxing the non-somatic variant filtering thresholds from paired germline (TMB_Germline), to ascending variant MAFs in 1000G and ExAC (TMB_DB0: MAF = 0; TMB_DB0.001: MAF ≤ 0.1%; TMB_DB0.01: MAF ≤ 1%). The last diagonal plot (bottom right) is the bar plot of the counts of 126 Black and 575 White patients included in the analyses. (2) The upper right panels of correlation values: the Pearson Correlation *r* values of TMBs across patients. The black numbers are the *r* values of all 701 patients. The last columns of the upper right panels are the box plots of the TMBs from different filtering criteria. (3) The lower left panels of dot plots illustrate the individual TMB values per patient; and the last row of the lower left panels are the jittered-point bar plots of individual TMB values.

The gold standard for identifying somatic mutations is to filter out non-somatic variants using patient-paired germline DNA sequencing data. TMBs estimated using this standard approach were comparable between tumors from Black and White patients (TMB of 6.09 ± 0.21, mean ± S.E, in Black patients; 5.47 ± 0.10 in White patients) (Table 1). However, when public variant DBs of 1000 Genomes Project (1000G) and Exome Aggregation Consortium (ExAC) were used to filter non-somatic variants, the TMB estimates were significantly inflated (Figs. 1a and 2, and Table 1) in tumors from both Black and White patients, with inverse correlations between TMBs and population minor allele frequency (MAF) threshold stringencies. In addition, the TMBs in the tumors from Black patients were inflated significantly higher compared to those in the tumors from White patients, with a race:filtering interaction *p* < 2e−16 by two-way ANOVA. When TMB was calculated from 1059 cancer-related genes only, similar observations were made (Fig. 1b). Importantly, while the TMBs across patients correlated well between values using different population MAF thresholds of the public DBs for non-somatic variant filtering (Fig. 2, row 2 columns 3–4, and row 3 column 4), the TMB from paired germline filtering had much lower correlations with TMB from public DB filtering of any threshold (Fig. 2, row 1, columns 2–4). This finding suggests that the impact of tumor-only sequencing on TMB estimates varied substantially from patient to patient.

**Table 1:** TMB (mutations/Mb) estimate in patients using different criteria of non-somatic variant filtering.

Filtering criteria	Mean (standard error)	
	Black	White
Germline paired	6.086 (0.209)	5.468 (0.104)
MAF = 0	7.858 (0.075)	7.428 (0.052)
MAF ≤ 0.001	12.116 (0.089)	10.099 (0.061)
MAF ≤ 0.01	22.425 (0.184)	13.501 (0.070)

In addition to 1000G and ExAC, ESP6500 (ref. ^7^) DB was also tested for variant filtering (Supplementary File 2 and Supplementary Fig. 3), which also resulted in more significantly inflated TMBs in Black compared to White patients.

TMB-H status is now an FDA-approved patient selection biomarker for ICI therapy. Because the collection of patient-matched germline samples is still not a common practice in clinic, TMBs are routinely estimated using tumor-only sequencing which led to significantly inflated TMB estimates^4^. Here we demonstrate that TMB inflations are racially disparate with significantly higher inflated TMBs in the tumors from Black patients due to the under-representation of minority groups in public variant DBs for variant filtering, regardless whether all (Figs. 1 and 2) or race-specific (Supplementary File 2 and Supplementary Fig. 2) variants from public DBs were used. If the currently approved TMB-H threshold of ≥10 mutations/Mb was hypothetically applied to the patients studied here, significantly higher numbers of Black patients would have been inappropriately selected to receive ICI therapy. It needs to be emphasized that we performed this proof-of-principle study in patients with multiple myeloma; however, the findings of racially disparate TMB inflation might be generalizable to all cancer types. Accurate TMB estimate is particularly important in cancers currently treated by ICIs including breast, bladder, cervical, colon, head and neck, liver, lung, renal cell, stomach, and rectal cancers, as well as Hodgkin lymphoma, melanoma, and any other solid tumor that is not able to repair errors during DNA replication (https://www.cancer.gov/about-cancer/treatment/types/immunotherapy/checkpoint-inhibitors).

In addition, the inflated TMBs are likely relevant for other ethnic groups including Asians, Pacific Islanders, and other under-represented groups.

Mutations are a surrogate for neoantigens. Not all mutations are expressed, presented by MHC proteins, and recognized by the adaptive immune system for elimination. Furthermore, frameshifts^8^ or chromosomal rearrangements^9^ may result in more potent neoantigens than single nucleotide substitutions. TMB is an appropriate step toward the application of immunotherapy; however, additional work that helps us to understand the quality of mutations rather than the quantity may refine this approach^10^. Just as mutations are a surrogate for neoantigens, self-reported race is a poor surrogate for geographical ancestry and individual polymorphisms. Even though race is a social construct that fails to encompass all the complexities of one’s identity and social determinants of health, we felt race was important to investigate in the context of TMB given the use of population DBs for variant filtering.

Clinicians who rely on TMB calculated from tumor-only sequencing as a biomarker for patient selection to receive ICIs need to be aware of the potential for inflated TMB values, especially in patients who are under-represented in the public genetic variant DBs.

## Methods

### Dataset and subject selection

Genomic sequencing data of participants in the Multiple Myeloma Research Foundation (MMRF) CoMMpass^SM^ study were used (https://themmrf.org/we-are-curing-multiple-myeloma/mmrf-commpass-study/). The anonymized tumor and patient-matched germline exome data were obtained from dbGAP (accession phs000748). The clinical and demographic features of each patient were downloaded from the MMRF Researcher Gateway (https://research.themmrf.org/). Of 701 patients with newly diagnosed multiple myeloma, 575 (82%) self-identified as White and 126 (18%) self-identified as Black.

### Variant calling

Sequencing reads of the tumor and patient-matched germline exomes were downloaded from the Sequencing Read Archive (SRA). The CoMMpass^SM^ study provided the list of somatic mutations only. In order to examine the impact of different variant filtering strategies on the calculation of patient-specific TMB, we performed variant calling in individual tumor and germline exomes without pairing. The paired-end sequencing reads were aligned to Human Reference Genome Build GRCh38 using BWA-MEM version 0.7.10^11^, and Broad’s best practice workflow for short variant discovery were followed (https://gatk.broadinstitute.org/hc/en-us/articles/360035894711-About-the-GATK-Best-Practices). Briefly, after read alignment, marking duplicates, and recalibration of base quality scores, variant calling per sample was performed using HaplotypeCaller^12^, and the joint calling of the consolidated GVCFs were carried out to obtain a list of raw SNVs and INDELs. The Variant Quality Score Recalibration (VQSR) model from Broad’s Genome Analysis Toolkit (GATK)^12^ was used to rank variants. Variants that passed the VQSR quality threshold were annotated using BioR^13^ to obtain functional impact of variants and their population allele frequencies in various DBs including the 1000G phase 3 (https://www.internationalgenome.org/), and the ExAC^5^.

### Filtering approaches

Somatic mutations were identified using four filtering criteria: (1) excluding variants in patient-matched germline exome; (2) excluding variants with MAF ≥ 1% in 1000G or ExAC DBs; (3) excluding variants with MAF ≥ 0.1% in 1000G or ExAC; and (4) excluding all variants reported by 1000G or ExAC. The TMBs were calculated as number of protein-altering or nonsynonymous somatic mutations (CAVA^14^ impact score of “Moderate” or “High”) per Mb of coding regions. The CoMMpass^SM^ study used the Agilent Human All Exon V5+UTR exome capture kit with a targeted region size of 75 Mb. TMBs were also calculated using 1059 cancer-related genes as defined by OncoKB (https://www.oncokb.org/, Fig. 1b). Total exon length of these cancer genes is 7 Mb.

### Statistical testing

The TMB values were approximately normal (Supplementary File 2 and Supplementary Fig. 1). For each of the four filtering criteria, the comparisons of TMB between Black and White individuals were performed using Student’s *t*-test. Two-way ANOVA model was used to measure the interaction between two independent variables: race (Black or White) and filtering criteria (four criteria as described above) (TMB ~ race + filtering + race:filtering).

## Supplementary information

Supplemental

Data

Data

## Data Availability

The data generated and analyzed during this study are described in the following *figshare* data record: 10.6084/m9.figshare.13664540. The exome sequencing data of tumor (CD138+ bone marrows) and patient-paired germline samples can be obtained by controlled access from *dbGAP* under accession https://identifiers.org/dbgap:phs000748. The self-reported race and other clinical characters of the patients can be accessed from the Multiple Myeloma Research Foundation Researcher Gateway (https://research.themmrf.org/). Additionally, the data underlying the figures, table, and supplementary file, as well as Supplementary File 1 (Excel spreadsheet), are shared as part of the *figshare* data record in the files “Supporting_Data.xlsx” and “Supplemental File 1.xlsx”.
