# Supplementary material for: Inflation of tumor mutation burden by tumor-only sequencing in under-represented groups
Source: NPJ Precis Oncol. 2021 Mar 19;5:22. doi: 10.1038/s41698-021-00164-5 (PMC7979755; doi:10.1038/s41698-021-00164-5)
Supplement: Supplementary file 1 — Supplemental [file 41698_2021_164_MOESM1_ESM.pdf]

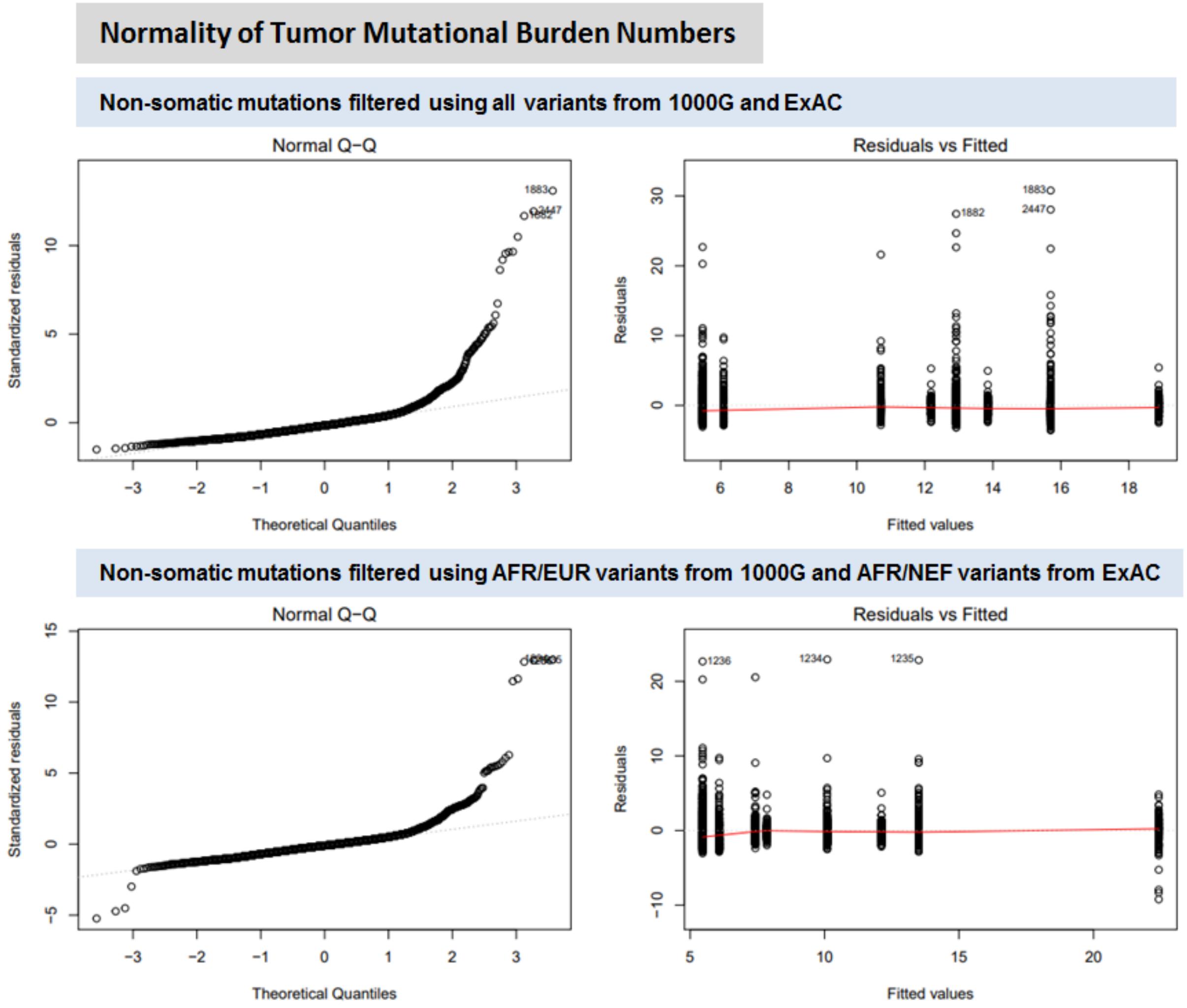

Supplementary Figure 1: Q-Q plot and Residual plot of Tumor Mutational Burden (TMB) values, as calculated as "number of protein-altering somatic mutations per Mb of coding regions". All TMB values from four different filtering criteria were included: : (1) excluding variants in patient-matched germline exome; (2) excluding variants with minor allele frequency (MAF) ≥ 1% in 1000G or ExAC DBs; (3) excluding variants with MAF ≥ 0.1% in 1000G or ExAC; and (4) excluding all variants reported by 1000G or ExAC. The upper panel figures illustrate the TMB normality when non-somatic mutations were filtered using germline or all variants in 1000G/ExAC. The lower panel figures used TMB values when non-somatic mutations were filtered using germline or race-specific variants in 1000G/ExAC

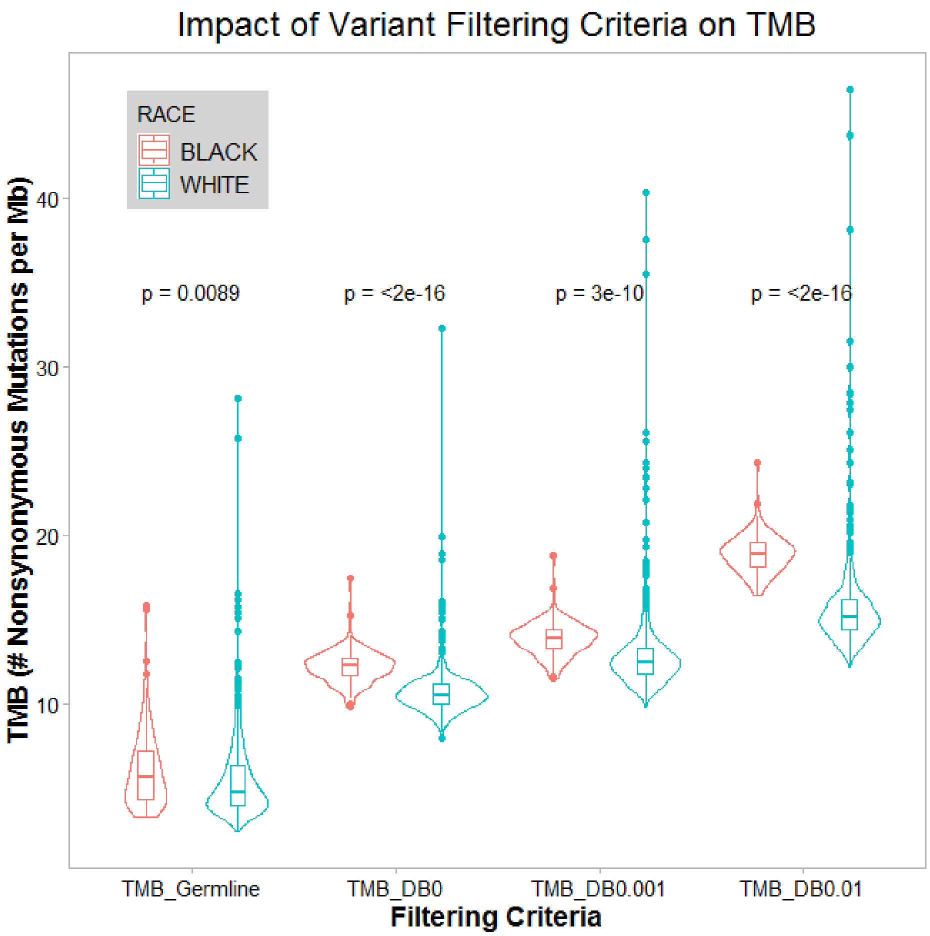

Supplementary Figure 2: Violin and Box Plot of Tumor Mutational Burdens (TMB), and Comparisons of TMBs Calculated from All Protein-Coding Genes in Black and White Patients from Four Variant Filtering Criteria. Note that variant MAF of AFR (African) population from 1000G and ExAC were used to filter variants in Black individuals, while MAF of EUR (European) population in 1000G and NFE (Non-Finnish European) in ExAC were used to filter variants in White patients. The TMB values were calculated as number of nonsynonymous mutations per Mb of coding regions. Four criteria were applied to identify patient-specific somatic mutations: (1) TMB_Germline: excluding variants in patient-matched germline exome; (2) TMB_DB0: excluding all variants reported by 1000G or ExAC; (3) TMB_DB0.001: excluding variants with MAF ≥ 0.1% in 1000G or ExAC; and (4) TMB_DB0.01: excluding variants with minor allele frequency (MAF) ≥ 1% in 1000G or ExAC DBs. The violin and box plots in red are TMB values from Black patients, and blue are from White Patients. Note that the 1000 Genome Project uses self-reported race, and ExAC's race definitions are computed from individual genotypes.

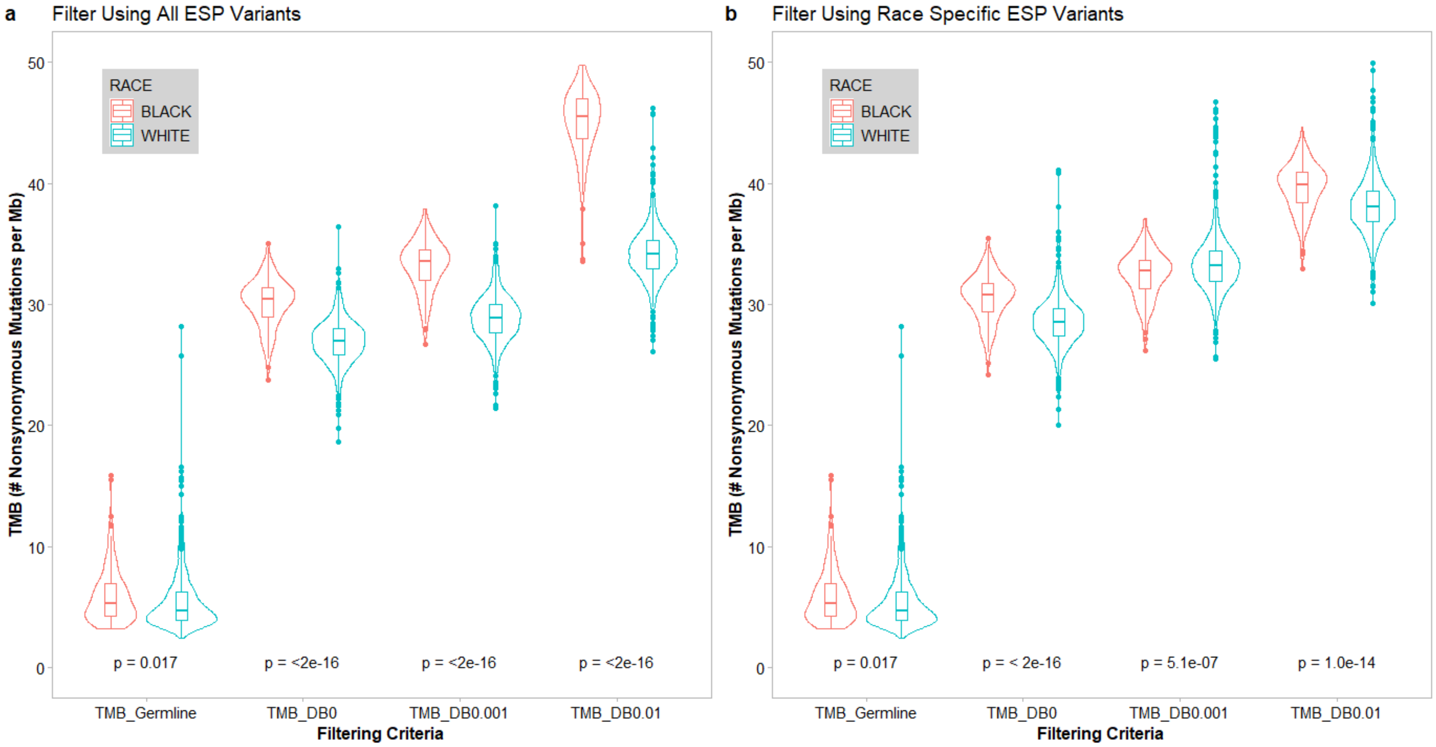

Supplementary Figure 3: Violin and Box Plot of Tumor Mutational Burdens (TMB), and Comparisons of TMBs Calculated from All Protein-Coding Genes in Black and White Patients from Four Variant Filtering Criteria Using ESP6500 Database. (a) variant MAF of all ESP6500 variants were used; (b) MAFs of AA (African American, n=2,217) population from ESP6500 were used to filter variants in Black individuals, and MAFs of EA (European American, n=4,298) population in ESP6500 were used to filter variants in White patients. The violin and box plots in red are TMB values from Black patients, and blue are from White Patients.

Hugo Symbol	Entrez Gene ID	Isoform	RefSeq
ABI1	10006		
ABL1	25	ENST00000318560	NM_005157.4
ABL2	27	ENST00000502732	NM_007314.3
ACKR3	57007		
ACSL3	2181		
ACSL6	23305		
ACTB	60		
ACTG1	71	ENST00000331925	NM_001199954.1
ACVR1	90	ENST00000263640	NM_001111067.2
ACVR1B	91		
ACVR2A	92		
ADGRA2	25960		
AFDN	4301		
AFF1	4299		
AFF3	3899		
AFF4	27125		
AGO1	26523	ENST00000373204	NM_012199.2
AGO2	27161	ENST00000220592	NM_012154.3
AJUBA	84962	ENST00000262713	NM_032876.5
AKT1	207	ENST00000349310	NM_001014431.1
AKT2	208	ENST00000392038	NM_001626.4
AKT3	10000	ENST00000263826	NM_005465.4
ALB	213	ENST00000295897	NM_000477.5
ALK	238	ENST00000389048	NM_004304.4
ALOX12B	242	ENST00000319144	NM_001139.2
AMER1	139285	ENST00000330258	NM_152424.3
ANKRD11	29123	ENST00000301030	NM_013275.5
APC	324	ENST00000257430	NM_000038.5
APH1A	51107		
APLNR	187	ENST00000257254	NM_005161.4
APOBEC3B	9582		
AR	367	ENST00000374690	NM_000044.3
ARAF	369	ENST00000377045	NM_001654.4
ARFRP1	10139		
ARHGAP26	23092		
ARHGAP35	2909	ENST00000404338	NM_004491.4
ARHGEF12	23365		
ARHGEF28	64283	ENST00000426542	NM_001177693.1
ARID1A	8289	ENST00000324856	NM_006015.4
ARID1B	57492	ENST00000346085	NM_020732.3
ARID2	196528	ENST00000334344	NM_152641.2
ARID3A	1820	ENST00000263620	NM_005224.2
ARID3B	10620	ENST00000622429	NM_001307939.1
ARID3C	138715	ENST00000378909	NM_001017363.1
ARID4A	5926	ENST00000355431	NM_002892.3
ARID4B	51742	ENST00000264183	NM_001206794.1
ARID5A	10865	ENST00000357485	NM_212481.1
ARID5B	84159	ENST00000279873	NM_032199.2
ARNT	405		
ASMTL	8623		
ASPSCR1	79058		
ASXL1	171023	ENST00000375687	NM_015338.5
ASXL2	55252	ENST00000435504	NM_018263.4

ATF1	466	ENST00000262053	NM_005171.4
ATG5	9474		
ATIC	471		
ATM	472	ENST00000278616	NM_000051.3
ATP1A1	476		
ATP2B3	492		
ATP6AP1	537	ENST00000369762	NM_001183.5
ATP6V1B2	526	ENST00000276390	NM_001693.3
ATR	545	ENST00000350721	NM_001184.3
ATRX	546	ENST00000373344	NM_000489.3
ATXN2	6311	ENST00000377617	NM_002973.3
ATXN7	6314	ENST00000295900	NM_000333.3
AURKA	6790	ENST00000312783	NM_003600.2
AURKB	9212	ENST00000585124	NM_004217.3
AXIN1	8312	ENST00000262320	NM_003502.3
AXIN2	8313	ENST00000307078	NM_004655.3
AXL	558	ENST00000301178	NM_021913.4
B2M	567	ENST00000558401	NM_004048.2
BABAM1	29086	ENST00000359435	NM_001033549.1
BACH2	60468	ENST00000257749	NM_001170794.1
BAP1	8314	ENST00000460680	NM_004656.3
BARD1	580	ENST00000260947	NM_000465.2
BAX	581		
BBC3	27113	ENST00000449228	NM_001127240.2
BCL10	8915	ENST00000370580	NM_003921.4
BCL11A	53335		
BCL11B	64919	ENST00000357195	NM_138576.3
BCL2	596	ENST00000333681	NM_000633.2
BCL2L1	598	ENST00000307677	NM_138578.1
BCL2L11	10018	ENST00000393256	NM_138621.4
BCL2L2	599		
BCL3	602		
BCL6	604	ENST00000232014	NM_001706.4
BCL7A	605		
BCL9	607	ENST00000234739	NM_004326.3
BCL9L	283149		
BCOR	54880	ENST00000378444	NM_001123385.1
BCORL1	63035	ENST00000218147	
BCR	613	ENST00000305877	NM_004327.3
BIRC3	330	ENST00000263464	NM_182962.2
BLM	641	ENST00000355112	NM_000057.2
BMPR1A	657	ENST00000372037	NM_004329.2
BRAF	673	ENST00000288602	NM_004333.4
BRCA1	672	ENST00000357654	NM_007294.3
BRCA2	675	ENST00000380152	NM_000059.3
BRD3	8019		
BRD4	23476	ENST00000263377	NM_058243.2
BRIP1	83990	ENST00000259008	NM_032043.2
BRSK1	84446		
BTG1	694	ENST00000256015	NM_001731.2
BTG2	7832		
BTK	695	ENST00000308731	NM_000061.2
BTLA	151888		
BUB1B	701		

C2orf44	80304		
CACNA1D	776		
CAD	790		
CALR	811	ENST00000316448	NM_004343.3
CAMTA1	23261		
CANT1	124583		
CARD11	84433	ENST00000396946	NM_032415.4
CARM1	10498	ENST00000327064	NM_199141.1
CARS	833		
CASC5	57082		
CASP8	841	ENST00000358485	NM_001080125.1
CBFA2T3	863		
CBFB	865	ENST00000412916	NM_022845.2
CBL	867	ENST00000264033	NM_005188.3
CBLB	868		
CBLC	23624		
CCDC6	8030		
CCN6	8838		
CCNB1IP1	57820		
CCNB3	85417	ENST00000276014	NM_033031.2
CCND1	595	ENST00000227507	NM_053056.2
CCND2	894	ENST00000261254	NM_001759.3
CCND3	896	ENST00000372991	NM_001760.3
CCNE1	898	ENST00000262643	NM_001238.2
CCT6B	10693		
CD22	933		
CD274	29126	ENST00000381577	NM_014143.3
CD276	80381	ENST00000318443	NM_001024736.1
CD28	940	ENST00000324106	NM_006139.3
CD36	948		
CD58	965	ENST00000369489	NM_001779.2
CD70	970		
CD74	972		
CD79A	973	ENST00000221972	NM_001783.3
CD79B	974	ENST00000392795	NM_001039933.1
CDC42	998	ENST00000344548	NM_001791.3
CDC73	79577	ENST00000367435	NM_024529.4
CDH1	999	ENST00000261769	NM_004360.3
CDH11	1009		
CDK12	51755	ENST00000447079	NM_016507.2
CDK4	1019	ENST00000257904	NM_000075.3
CDK6	1021	ENST00000265734	NM_001145306.1
CDK8	1024	ENST00000381527	NM_001260.1
CDKN1A	1026	ENST00000244741	NM_078467.2
CDKN1B	1027	ENST00000228872	NM_004064.3
CDKN2A	1029	ENST00000304494	NM_000077.4
CDKN2B	1030	ENST00000276925	NM_004936.3
CDKN2C	1031	ENST00000262662	NM_078626.2
CDX2	1045		
CEBPA	1050	ENST00000498907	NM_004364.3
CENPA	1058	ENST00000335756	NM_001809.3
CHCHD7	79145		
CHD2	1106		
CHD4	1108		

CHEK1	1111	ENST00000428830	NM_001274.5
CHEK2	11200	ENST00000328354	NM_007194.3
CHIC2	26511		
CHN1	1123		
CIC	23152	ENST00000575354	NM_015125.3
CIITA	4261	ENST00000324288	
CKS1B	1163		
CLIP1	6249		
CLP1	10978		
CLTC	1213		
CLTCL1	8218		
CMTR2	55783	ENST00000338099	NM_001099642.1
CNBP	7555		
CNOT3	4849		
CNTRL	11064		
COL1A1	1277		
COL2A1	1280		
CPS1	1373		
CRBN	51185	ENST00000231948	NM_016302.3
CREB1	1385	ENST00000432329	NM_134442.3
CREB3L1	90993		
CREB3L2	64764		
CREBBP	1387	ENST00000262367	NM_004380.2
CRKL	1399	ENST00000354336	NM_005207.3
CRLF2	64109	ENST00000381566	NM_022148.2
CRTC1	23373		
CRTC3	64784		
CSDE1	7812	ENST00000438362	NM_001242891.1
CSF1	1435		
CSF1R	1436	ENST00000286301	NM_005211.3
CSF3R	1441	ENST00000361632	NM_000760.3
CTCF	10664	ENST00000264010	NM_006565.3
CTLA4	1493	ENST00000302823	NM_005214.4
CTNNA1	1495		
CTNNB1	1499	ENST00000349496	NM_001904.3
CTR9	9646	ENST00000361367	NM_014633.4
CUL3	8452	ENST00000264414	NM_003590.4
CUL4A	8451		
CUX1	1523	ENST00000292535	NM_181552.3
CXCR4	7852	ENST00000241393	NM_003467.2
CXORF67	340602		
CYLD	1540	ENST00000398568	NM_001042355.1
CYP17A1	1586		
CYP19A1	1588	ENST00000260433	NM_000103.3
CYSLTR2	57105	ENST00000282018	NM_020377.2
DAXX	1616	ENST00000374542	NM_001141970.1
DCTN1	1639		
DCUN1D1	54165	ENST00000292782	NM_020640.2
DDB2	1643		
DDIT3	1649	ENST00000346473	NM_001195057.1
DDR1	780		
DDR2	4921	ENST00000367921	NM_006182.2
DDX10	1662		
DDX3X	1654	ENST00000399959	NM_001356.4

DDX4	54514	ENST00000505374	NM_024415.2
DDX41	51428	ENST00000507955	NM_016222.2
DDX5	1655		
DDX6	1656		
DEK	7913	ENST00000397239	NM_003472.3
DICER1	23405	ENST00000343455	NM_030621.3
DIS3	22894	ENST00000377767	NM_014953.3
DKK1	22943	ENST00000373970	NM_012242.2
DKK2	27123	ENST00000285311	NM_014421.2
DKK3	27122	ENST00000326932	NM_001018057.1
DKK4	27121	ENST00000220812	NM_014420.2
DNAJB1	3337	ENST00000254322	NM_006145.1
DNM2	1785		
DNMT1	1786	ENST00000340748	NM_001379.2
DNMT3A	1788	ENST00000264709	NM_022552.4
DNMT3B	1789	ENST00000328111	NM_006892.3
DOT1L	84444	ENST00000398665	NM_032482.2
DROSHA	29102	ENST00000344624	NM_013235.4
DTX1	1840	ENST00000257600	NM_004416.2
DUSP2	1844		
DUSP22	56940	ENST00000344450	NM_020185.4
DUSP4	1846	ENST00000240100	NM_001394.6
DUSP9	1852		
E2F3	1871	ENST00000346618	NM_001949.4
EBF1	1879		
ECT2L	345930	ENST00000367682	NM_001077706.2
EED	8726	ENST00000263360	NM_003797.3
EGFL7	51162	ENST00000308874	NM_201446.2
EGFR	1956	ENST00000275493	NM_005228.3
EGR1	1958	ENST00000239938	NM_001964.2
EIF1AX	1964	ENST00000379607	NM_001412.3
EIF3E	3646		
EIF4A2	1974	ENST00000323963	NM_001967.3
EIF4E	1977	ENST00000280892	NM_001130678.1
ELF3	1999	ENST00000359651	NM_004433.4
ELF4	2000		
ELK4	2005		
ELL	8178		
ELMSAN1	91748	ENST00000286523	NM_001043318.2
ELN	2006		
ELP2	55250		
EML4	27436		
EMSY	56946		
EP300	2033	ENST00000263253	NM_001429.3
EP400	57634	ENST00000389561	NM_015409.3
EPAS1	2034	ENST00000263734	NM_001430.4
EPCAM	4072	ENST00000263735	NM_002354.2
EPHA3	2042	ENST00000336596	NM_005233.5
EPHA5	2044	ENST00000273854	NM_004439.5
EPHA7	2045	ENST00000369303	NM_004440.3
EPHB1	2047	ENST00000398015	NM_004441.4
EPHB4	2050		
EPOR	2057	ENST00000222139	NM_000121.3
EPS15	2060		

ERBB2	2064	ENST00000269571	NM_004448.2
ERBB3	2065	ENST00000267101	NM_001982.3
ERBB4	2066	ENST00000342788	NM_005235.2
ERC1	23085		
ERCC2	2068	ENST00000391945	NM_000400.3
ERCC3	2071	ENST00000285398	NM_000122.1
ERCC4	2072	ENST00000311895	NM_005236.2
ERCC5	2073	ENST00000355739	NM_000123.3
ERF	2077	ENST00000222329	NM_006494.2
ERG	2078	ENST00000288319	NM_182918.3
ERRFI1	54206	ENST00000377482	NM_018948.3
ESCO2	157570	ENST00000305188	NM_001017420.2
ESR1	2099	ENST00000206249	NM_001122740.1
ETAA1	54465	ENST00000272342	NM_019002.3
ETNK1	55500	ENST00000266517	NM_018638.4
ETS1	2113		
ETV1	2115	ENST00000405192	NM_001163147.1
ETV4	2118	ENST00000319349	NM_001079675.2
ETV5	2119	ENST00000306376	NM_004454.2
ETV6	2120	ENST00000396373	NM_001987.4
EWSR1	2130	ENST00000397938	NM_005243.3
EXOSC6	118460		
EXT1	2131		
EXT2	2132		
EZH1	2145	ENST00000428826	NM_001991.3
EZH2	2146	ENST00000320356	NM_004456.4
EZR	7430		
FAF1	11124		
FAM175A	84142	ENST00000321945	NM_139076.2
FAM46C	54855	ENST00000369448	NM_017709.3
FAM58A	92002	ENST00000406277	NM_152274.4
FANCA	2175	ENST00000389301	NM_000135.2
FANCC	2176	ENST00000289081	NM_000136.2
FANCD2	2177	ENST00000383807	NM_001018115.1
FANCE	2178		
FANCF	2188		
FANCG	2189		
FANCL	55120		
FAS	355	ENST00000355740	NM_000043.4
FAT1	2195	ENST00000441802	NM_005245.3
FAT4	79633		
FBXO11	80204	ENST00000403359	NM_001190274.1
FBXO31	79791		
FBXW7	55294	ENST00000281708	NM_033632.3
FCGR2B	2213		
FCRL4	83417		
FES	2242		
FEV	54738	ENST00000295727	NM_017521.2
FGF10	2255		
FGF12	2257		
FGF14	2259		
FGF19	9965	ENST00000294312	NM_005117.2
FGF23	8074		
FGF3	2248	ENST00000334134	NM_005247.2

FGF4	2249	ENST00000168712	NM_002007.2
FGF6	2251		
FGFR1	2260	ENST00000425967	NM_001174067.1
FGFR1OP	11116		
FGFR2	2263	ENST00000358487	NM_000141.4
FGFR3	2261	ENST00000260795	NM_000142.4
FGFR4	2264	ENST00000292408	NM_213647.1
FH	2271	ENST00000366560	NM_000143.3
FHIT	2272		
FIP1L1	81608		
FLCN	201163	ENST00000285071	NM_144997.5
FLI1	2313	ENST00000527786	NM_002017.4
FLT1	2321	ENST00000282397	NM_002019.4
FLT3	2322	ENST00000241453	NM_004119.2
FLT4	2324	ENST00000261937	NM_182925.4
FLYWCH1	84256		
FNBP1	23048		
FOXA1	3169	ENST00000250448	NM_004496.3
FOXF1	2294	ENST00000262426	NM_001451.2
FOXL2	668	ENST00000330315	NM_023067.3
FOXO1	2308	ENST00000379561	NM_002015.3
FOXO3	2309		
FOXO4	4303		
FOXP1	27086	ENST00000318789	NM_001244814.1
FRS2	10818		
FSTL3	10272		
FUBP1	8880	ENST00000370768	NM_003902.3
FURIN	5045	ENST00000268171	NM_001289823.1
FUS	2521		
FYN	2534	ENST00000368678	NM_153047.3
GAB1	2549	ENST00000262994	NM_002039.3
GAB2	9846	ENST00000361507	NM_080491.2
GABRA6	2559		
GADD45B	4616		
GAS7	8522		
GATA1	2623	ENST00000376670	NM_002049.3
GATA2	2624	ENST00000341105	NM_032638.4
GATA3	2625	ENST00000346208	NM_002051.2
GATA4	2626		
GATA6	2627		
GID4	79018		
GLI1	2735	ENST00000228682	NM_005269.2
GMPS	8833		
GNA11	2767	ENST00000078429	NM_002067.2
GNA12	2768	ENST00000275364	NM_007353.2
GNA13	10672	ENST00000439174	NM_006572.5
GNAQ	2776	ENST00000286548	NM_002072.3
GNAS	2778	ENST00000371085	NM_000516.4
GNB1	2782	ENST00000378609	NM_001282539.1
GOLGA5	9950		
GOPC	57120		
GPC3	2719		
GPHN	10243		
GPS2	2874	ENST00000380728	NM_004489.4

GREM1	26585	ENST00000300177	NM_013372.6
GRIN2A	2903	ENST00000330684	NM_001134407.1
GRM3	2913		
GSK3B	2932	ENST00000316626	NM_002093.3
GTF2I	2969	ENST00000324896	NM_032999.3
GTSE1	51512		
H3F3A	3020	ENST00000366813	NM_002107.4
H3F3AP4	440926	ENST00000316450	null
H3F3B	3021	ENST00000254810	NM_005324.3
H3F3C	440093	ENST00000340398	NM_001013699.2
HDAC1	3065	ENST00000373548	NM_004964.2
HDAC4	9759	ENST00000345617	NM_006037.3
HDAC7	51564	ENST00000427332	XM_011538481.1
HERPUD1	9709		
HEY1	23462		
HGF	3082	ENST00000222390	NM_000601.4
HIF1A	3091	ENST00000337138	NM_001530.3
HIP1	3092		
HIRA	7290		
HIST1H1B	3009	ENST00000331442	NM_005322.2
HIST1H1C	3006	ENST00000343677	NM_005319.3
HIST1H1D	3007	ENST00000244534	NM_005320.2
HIST1H1E	3008	ENST00000304218	NM_005321.2
HIST1H2AC	8334	ENST00000314088	NM_003512.3
HIST1H2AG	8969	ENST00000358739	NM_003509.2
HIST1H2AL	8332	ENST00000357320	NM_003511
HIST1H2AM	8336	ENST00000359611	NM_003514
HIST1H2BC	8347	ENST00000314332	NM_003518.3
HIST1H2BD	3017	ENST00000289316	NM_021063.3
HIST1H2BG	8339	ENST00000244601	NM_003518
HIST1H2BJ	8970	ENST00000339812	NM_021058.3
HIST1H2BK	85236	ENST00000356950	NM_080593.2
HIST1H2BO	8348	ENST00000616182	NM_003527.4
HIST1H3A	8350	ENST00000357647	NM_003529.2
HIST1H3B	8358	ENST00000244661	NM_003537.3
HIST1H3C	8352	ENST00000540144	NM_003531.2
HIST1H3D	8351	ENST00000356476	NM_003530.4
HIST1H3E	8353	ENST00000360408	NM_003532.2
HIST1H3F	8968	ENST00000446824	NM_021018.2
HIST1H3G	8355	ENST00000305910	NM_003534.2
HIST1H3H	8357	ENST00000369163	NM_003536.2
HIST1H3I	8354	ENST00000328488	NM_003533.2
HIST1H3J	8356	ENST00000359303	NM_003535.2
HIST1H4I	8294		
HIST2H3A	333932	ENST00000331491	NM_001005464.2
HIST2H3C	126961	ENST00000369158	NM_021059.2
HIST2H3D	653604	ENST00000331491	NM_001123375.2
HIST3H3	8290	ENST00000366696	NM_003493.2
HLA-A	3105	ENST00000376809	NM_001242758.1
HLA-B	3106	ENST00000412585	NM_005514.6
HLA-C	3107	ENST00000376228	NM_002117.5
HLF	3131		
HMGA1	3159		
HMGA2	8091		

HNF1A	6927	ENST00000257555	NM_000545.5
HNRNPA2B1	3181		
HOOK3	84376		
HOXA11	3207		
HOXA13	3209		
HOXA3	3200		
HOXA9	3205		
HOXB13	10481	ENST00000290295	NM_006361.5
HOXC11	3227		
HOXC13	3229		
HOXD11	3237		
HOXD13	3239		
HRAS	3265	ENST00000311189	NM_001130442.1
HSD3B1	3283		
HSP90AA1	3320		
HSP90AB1	3326		
ICK	22858		
ICOSLG	23308	ENST00000407780	NM_015259.4
ID3	3399	ENST00000374561	NM_002167.4
IDH1	3417	ENST00000345146	NM_005896.2
IDH2	3418	ENST00000330062	NM_002168.2
IFNGR1	3459	ENST00000367739	NM_000416.2
IGF1	3479	ENST00000307046	NM_001111285.1
IGF1R	3480	ENST00000268035	NM_000875.3
IGF2	3481	ENST00000434045	NM_001127598.1
IGH	3492		
IGK	50802		
IGL	3535		
IKBKB	3551		
IKBKE	9641	ENST00000367120	NM_014002.3
IKZF1	10320	ENST00000331340	ENST00000331340
IKZF2	22807		
IKZF3	22806	ENST00000346872	NM_012481.4
IL10	3586	ENST00000423557	NM_000572.2
IL2	3558		
IL21R	50615		
IL3	3562	ENST00000296870	NM_000588.3
IL6ST	3572		
IL7R	3575	ENST00000303115	NM_002185.3
INHA	3623	ENST00000243786	NM_002191.3
INHBA	3624	ENST00000242208	NM_002192.2
INPP4A	3631	ENST00000074304	NM_001134224.1
INPP4B	8821	ENST00000262992	NM_001101669.1
INPP5D	3635		
INPPL1	3636	ENST00000298229	NM_001567.3
INSR	3643	ENST00000302850	NM_000208.2
IRF1	3659	ENST00000245414	NM_002198.2
IRF2	3660		
IRF4	3662	ENST00000380956	NM_002460.3
IRF8	3394	ENST00000268638	NM_002163.2
IRS1	3667	ENST00000305123	NM_005544.2
IRS2	8660	ENST00000375856	NM_003749.2
IRS4	8471		
ITK	3702		

JAK1	3716	ENST00000342505	NM_002227.2
JAK2	3717	ENST00000381652	NM_004972.3
JAK3	3718	ENST00000458235	NM_000215.3
JARID2	3720	ENST00000341776	NM_004973.3
JAZF1	221895		
JUN	3725	ENST00000371222	NM_002228.3
KAT6A	7994	ENST00000265713	NM_006766.4
KAT6B	23522		
KBTBD4	55709	ENST00000395288	NM_016506.5
KCNJ5	3762		
KDM2B	84678		
KDM4C	23081		
KDM5A	5927	ENST00000399788	NM_001042603.1
KDM5C	8242	ENST00000375401	NM_004187.3
KDM6A	7403	ENST00000377967	NM_021140.2
KDR	3791	ENST00000263923	NM_002253.2
KDSR	2531		
KEAP1	9817	ENST00000171111	NM_203500.1
KEL	3792		
KIF5B	3799		
KIT	3815	ENST00000288135	NM_000222.2
KLF2	10365	ENST00000248071	NM_016270.2
KLF4	9314	ENST00000374672	NM_004235.4
KLF5	688	ENST00000377687	NM_001730.4
KLF6	1316		
KLHL6	89857		
KLK2	3817		
KMT2A	4297	ENST00000534358	NM_001197104.1
KMT2B	9757	ENST00000222270	NM_014727.1
KMT2C	58508	ENST00000262189	NM_170606.2
KMT2D	8085	ENST00000301067	NM_003482.3
KMT5A	387893	ENST00000330479	NM_020382.3
KNSTRN	90417	ENST00000249776	NM_033286.3
KRAS	3845	ENST00000256078	NM_033360.2
KSR2	283455	ENST00000339824	
KTN1	3895		
LASP1	3927		
LATS1	9113	ENST00000253339	NM_004690.3
LATS2	26524	ENST00000382592	NM_014572.2
LCK	3932	ENST00000336890	NM_001042771.2
LCP1	3936		
LEF1	51176		
LIFR	3977		
LMNA	4000		
LMO1	4004	ENST00000335790	NM_002315.2
LMO2	4005	ENST00000395833	NM_001142315.1
LPP	4026		
LRIG3	121227		
LRP1B	53353		
LRP5	4041	ENST00000294304	NM_001291902.1
LRP6	4040	ENST00000261349	NM_002336.2
LRRK2	120892		
LTB	4050	ENST00000376117	NM_002341.1
LTK	4058		

LYL1	4066		
LYN	4067	ENST00000519728	NM_002350.3
LZTR1	8216	ENST00000215739	NM_006767.3
MAD2L2	10459	ENST00000235310	NM_001127325.1
MAF	4094	ENST00000393350	NM_001031804.2
MAFB	9935	ENST00000373313	NM_005461.4
MAGED1	9500		
MALT1	10892	ENST00000348428	NM_006785.3
MAML2	84441		
MAP2K1	5604	ENST00000307102	NM_002755.3
MAP2K2	5605	ENST00000262948	NM_030662.3
MAP2K4	6416	ENST00000353533	NM_003010.3
MAP3K1	4214	ENST00000399503	NM_005921.1
MAP3K13	9175	ENST00000265026	NM_004721.4
MAP3K14	9020	ENST00000344686	NM_003954.3
MAP3K6	9064		
MAP3K7	6885		
MAPK1	5594	ENST00000215832	NM_002745.4
MAPK3	5595	ENST00000263025	NM_002746.2
MAPKAP1	79109	ENST00000265960	NM_001006617.1
MAX	4149	ENST00000358664	NM_002382.4
MBD6	114785	ENST00000355673	NM_052897.3
MCL1	4170	ENST00000369026	NM_021960.4
MDC1	9656	ENST00000376406	NM_014641.2
MDM2	4193	ENST00000462284	NM_002392.5
MDM4	4194	ENST00000367182	NM_002393.4
MDS2	259283		
MECOM	2122	ENST00000468789	NM_001105078.3
MED12	9968	ENST00000374080	NM_005120.2
MEF2B	100271849	ENST00000162023	NM_001145785.1
MEF2C	4208		
MEN1	4221	ENST00000337652	NM_000244.3
MERTK	10461		
MET	4233	ENST00000397752	NM_000245.2
MGA	23269	ENST00000219905	NM_001164273.1
MGAM	8972	ENST00000549489	NM_004668.2
MIB1	57534		
MITF	4286	ENST00000352241	NM_198159.2
MKI67	4288		
MKNK1	8569		
MLF1	4291		
MLH1	4292	ENST00000231790	NM_000249.3
MLLT1	4298	ENST00000252674	NM_005934.3
MLLT10	8028	ENST00000307729	NM_001195626.1
MLLT11	10962		
MLLT3	4300		
MLLT6	4302		
MN1	4330		
MNX1	3110		
MOB3B	79817	ENST00000262244	NM_024761.4
MPEG1	219972	ENST00000361050	NM_001039396.1
MPL	4352	ENST00000372470	NM_005373.2
MRE11A	4361	ENST00000323929	NM_005591.3
MRTFA	57591		

MSH2	4436	ENST00000233146	NM_000251.2
MSH3	4437	ENST00000265081	NM_002439.4
MSH6	2956	ENST00000234420	NM_000179.2
MSI1	4440	ENST00000257552	NM_002442.3
MSI2	124540	ENST00000284073	NM_138962.2
MSN	4478		
MST1	4485	ENST00000449682	NM_020998.3
MST1R	4486	ENST00000296474	NM_002447.2
MTAP	4507	ENST00000380172	NM_002451.3
MTCP1	4515		
MTOR	2475	ENST00000361445	NM_004958.3
MUC1	4582		
MUTYH	4595	ENST00000372115	NM_001048171.1
MYB	4602		
MYC	4609	ENST00000377970	NM_002467.4
MYCL	4610	ENST00000397332	NM_001033082.2
MYCN	4613	ENST00000281043	NM_005378.4
MYD88	4615	ENST00000396334	NM_002468.4
MYH11	4629		
MYH9	4627		
MYO18A	399687		
MYO5A	4644		
MYOD1	4654	ENST00000250003	NM_002478.4
NAB2	4665		
NACA	4666		
NADK	65220	ENST00000341426	NM_001198993.1
NBEAP1	606		
NBN	4683	ENST00000265433	NM_002485.4
NCOA1	8648		
NCOA2	10499		
NCOA3	8202	ENST00000371998	NM_181659.2
NCOA4	8031		
NCOR1	9611	ENST00000268712	NM_006311.3
NCOR2	9612	ENST00000405201	NM_006312.6
NCSTN	23385	ENST00000294785	NM_015331.2
NDRG1	10397		
NEGR1	257194	ENST00000357731	NM_173808.2
NF1	4763	ENST00000358273	NM_001042492.2
NF2	4771	ENST00000338641	NM_000268.3
NFATC2	4773		
NFE2	4778	ENST00000312156	NM_001136023.2
NFE2L2	4780	ENST00000397062	NM_006164.4
NFIB	4781		
NFKB2	4791		
NFKBIA	4792	ENST00000216797	NM_020529.2
NFKBIE	4794		
NIN	51199		
NKX2-1	7080	ENST00000354822	NM_001079668.2
NKX3-1	4824	ENST00000380871	NM_006167.3
NOD1	10392		
NONO	4841		
NOTCH1	4851	ENST00000277541	NM_017617.3
NOTCH2	4853	ENST00000256646	NM_024408.3
NOTCH3	4854	ENST00000263388	NM_000435.2

NOTCH4	4855	ENST00000375023	NM_004557.3
NPM1	4869	ENST00000296930	NM_002520.6
NR4A3	8013	ENST00000395097	NM_006981.3
NRAS	4893	ENST00000369535	NM_002524.4
NRG1	3084	ENST00000405005	NM_013964.3
NSD1	64324	ENST00000439151	NM_022455.4
NT5C2	22978	ENST00000343289	NM_001134373.2
NTHL1	4913	ENST00000219066	NM_002528.5
NTRK1	4914	ENST00000524377	NM_002529.3
NTRK2	4915	ENST00000277120	NM_006180.3
NTRK3	4916	ENST00000360948	NM_001012338.2
NUF2	83540	ENST00000271452	NM_031423.3
NUMA1	4926		
NUP214	8021		
NUP93	9688	ENST00000308159	NM_014669.4
NUP98	4928	ENST00000359171	XM_005252950.1
NUTM1	256646	ENST00000333756	XM_011521429.1
NUTM2A	728118		
NUTM2B	729262		
OLIG2	10215		
OMD	4958		
P2RY8	286530	ENST00000381297	NM_178129.4
PAFAH1B2	5049		
PAG1	55824		
PAK1	5058	ENST00000356341	NM_002576.4
PAK3	5063		
PAK5	57144	ENST00000353224	NM_177990.2
PALB2	79728	ENST00000261584	NM_024675.3
PARK2	5071	ENST00000366898	NM_004562.2
PARP1	142	ENST00000366794	NM_001618.3
PARP2	10038		
PARP3	10039		
PASK	23178		
PAX3	5077		
PAX5	5079	ENST00000358127	NM_016734.2
PAX7	5081		
PAX8	7849	ENST00000263334	NM_003466.3
PBRM1	55193	ENST00000394830	NM_018313.4
PBX1	5087		
PC	5091		
PCBP1	5093	ENST00000303577	NM_006196.3
PCLO	27445		
PCM1	5108		
PCSK7	9159		
PDCD1	5133	ENST00000334409	NM_005018.2
PDCD11	22984		
PDCD1LG2	80380	ENST00000397747	NM_025239.3
PDE4DIP	9659		
PDGFB	5155	ENST00000331163	NM_002608.2
PDGFRA	5156	ENST00000257290	NM_006206.4
PDGFRB	5159	ENST00000261799	NM_002609.3
PDK1	5163		
PDPK1	5170	ENST00000342085	NM_002613.4
PDS5B	23047	ENST00000315596	NM_015032.3

PER1	5187		
PGBD5	79605	ENST00000525115	NM_001258311.1
PGR	5241	ENST00000325455	NM_000926.4
PHF1	5252		
PHF6	84295	ENST00000332070	NM_001015877.1
PHOX2B	8929	ENST00000226382	NM_003924.3
PICALM	8301		
PIGA	5277	ENST00000333590	NM_002641.3
PIK3C2B	5287		
PIK3C2G	5288	ENST00000266497	NM_004570.4
PIK3C3	5289	ENST00000262039	NM_002647.2
PIK3CA	5290	ENST00000263967	NM_006218.2
PIK3CB	5291	ENST00000289153	NM_006219.2
PIK3CD	5293	ENST00000377346	NM_005026.3
PIK3CG	5294	ENST00000359195	NM_002649.2
PIK3R1	5295	ENST00000274335	NM_181523.2
PIK3R2	5296	ENST00000222254	NM_005027.3
PIK3R3	8503	ENST00000262741	NM_003629.3
PIM1	5292	ENST00000373509	NM_002648.3
PLAG1	5324		
PLCG1	5335	ENST00000373271	NM_182811.1
PLCG2	5336	ENST00000359376	NM_002661.3
PLK2	10769	ENST00000274289	NM_006622.3
PMAIP1	5366	ENST00000316660	NM_021127.2
PML	5371		
PMS1	5378	ENST00000441310	NM_000534.4
PMS2	5395	ENST00000265849	NM_000535.5
PNRC1	10957	ENST00000336032	NM_006813.2
POLD1	5424	ENST00000440232	NM_002691.3
POLE	5426	ENST00000320574	NM_006231.2
POLQ	10721		
POT1	25913	ENST00000357628	NM_015450.2
POU2AF1	5450		
POU5F1	5460		
PPARG	5468	ENST00000287820	NM_015869.4
PPFIBP1	8496		
PPM1D	8493	ENST00000305921	NM_003620.3
PPP1CB	5500		
PPP2R1A	5518	ENST00000322088	NM_014225.5
PPP2R2A	5520		
PPP4R2	151987	ENST00000356692	NM_174907.2
PPP6C	5537	ENST00000373547	NM_002721.4
PRCC	5546		
PRDM1	639	ENST00000369096	NM_001198.3
PRDM14	63978	ENST00000276594	NM_024504.3
PRDM16	63976		
PREX2	80243	ENST00000288368	NM_024870.2
PRF1	5551		
PRKACA	5566	ENST00000308677	NM_002730.3
PRKAR1A	5573	ENST00000358598	NM_212471.2
PRKCI	5584	ENST00000295797	NM_002740.5
PRKD1	5587	ENST00000331968	NM_002742.2
PRKDC	5591		
PRRX1	5396		

PRSS1	5644	ENST00000311737	NM_002769.4
PRSS8	5652		
PSIP1	11168		
PTCH1	5727	ENST00000331920	NM_000264.3
PTEN	5728	ENST00000371953	NM_000314.4
PTK6	5753		
PTK7	5754		
PTP4A1	7803	ENST00000370651	NM_003463.4
PTPN1	5770	ENST00000371621	NM_001278618.1
PTPN11	5781	ENST00000351677	NM_002834.3
PTPN13	5783		
PTPN2	5771	ENST00000309660	NM_002828.3
PTPN6	5777		
PTPRB	5787		
PTPRC	5788		
PTPRD	5789	ENST00000356435	NM_002839.3
PTPRK	5796		
PTPRO	5800		
PTPRS	5802	ENST00000357368	NM_002850.3
PTPRT	11122	ENST00000373198	NM_133170.3
QKI	9444		
RAB35	11021	ENST00000229340	NM_006861.6
RABEP1	9135		
RAC1	5879	ENST00000356142	NM_018890.3
RAC2	5880	ENST00000249071	NM_002872.4
RAD21	5885	ENST00000297338	NM_006265.2
RAD50	10111	ENST00000265335	NM_005732.3
RAD51	5888	ENST00000267868	NM_002875.4
RAD51B	5890	ENST00000487270	NM_133509.3
RAD51C	5889	ENST00000337432	NM_058216.2
RAD51D	5892	ENST00000335858	NM_133629.2
RAD52	5893	ENST00000358495	NM_134424.2
RAD54L	8438	ENST00000371975	NM_001142548.1
RAF1	5894	ENST00000251849	NM_002880.3
RALGDS	5900		
RANBP2	5903		
RAP1GDS1	5910		
RARA	5914	ENST00000254066	NM_000964.3
RASA1	5921	ENST00000274376	NM_002890.2
RASGEF1A	221002		
RB1	5925	ENST00000267163	NM_000321.2
RBM10	8241	ENST00000329236	NM_001204468.1
RBM15	64783	ENST00000369784	NM_022768.4
RECQL	5965	ENST00000421138	NM_032941.2
RECQL4	9401	ENST00000428558	ENST00000428558
REL	5966	ENST00000295025	NM_002908.2
RELN	5649	ENST00000428762	NM_005045.3
REST	5978	ENST00000309042	NM_001193508.1
RET	5979	ENST00000355710	NM_020975.4
RFWD2	64326	ENST00000367669	NM_022457.5
RHEB	6009	ENST00000262187	NM_005614.3
RHOA	387	ENST00000418115	NM_001664.2
RHOH	399		
RICTOR	253260	ENST00000357387	NM_152756.3

RIT1	6016	ENST00000368323	NM_006912.5
RMI2	116028		
RNF213	57674		
RNF217-AS1	7955		
RNF43	54894	ENST00000407977	NM_017763.4
ROBO1	6091	ENST00000464233	NM_002941.3
ROS1	6098	ENST00000368508	NM_002944.2
RPL10	6134		
RPL22	6146		
RPL5	6125		
RPN1	6184		
RPS6KA4	8986	ENST00000334205	NM_003942.2
RPS6KB2	6199	ENST00000312629	NM_003952.2
RPTOR	57521	ENST00000306801	NM_020761.2
RRAGC	64121	ENST00000373001	NM_022157.3
RRAS	6237	ENST00000246792	NM_006270.3
RRAS2	22800	ENST00000256196	NM_012250.5
RSPO2	340419		
RSPO3	84870		
RTEL1	51750	ENST00000508582	NM_032957.4
RUNX1	861	ENST00000300305	NM_001754.4
RUNX1T1	862	ENST00000265814	NM_001198626.1
RUNX2	860		
RXRA	6256	ENST00000481739	NM_002957.4
RYBP	23429	ENST00000477973	NM_012234.5
S1PR2	9294		
SALL4	57167		
SAMHD1	25939	ENST00000262878	NM_015474.3
SBDS	51119		
SCG5	6447	ENST00000300175	NM_001144757.1
SDC4	6385		
SDHA	6389	ENST00000264932	NM_004168.2
SDHAF2	54949	ENST00000301761	NM_017841.2
SDHB	6390	ENST00000375499	NM_003000.2
SDHC	6391	ENST00000367975	NM_003001.3
SDHD	6392	ENST00000375549	NM_003002.3
SEC31A	22872		
SEPTIN5	5413		
SEPTIN6	23157		
SEPTIN9	10801		
SERP2	387923		
SERPINB3	6317	ENST00000283752	NM_006919.2
SERPINB4	6318	ENST00000341074	NM_002974.3
SESN1	27244	ENST00000436639	NM_014454.2
SESN2	83667	ENST00000253063	NM_031459.4
SESN3	143686	ENST00000536441	NM_144665.3
SET	6418		
SETBP1	26040	ENST00000282030	NM_015559.2
SETD1A	9739	ENST00000262519	NM_014712.2
SETD1B	23067	ENST00000604567	XM_005253858.3
SETD2	29072	ENST00000409792	NM_014159.6
SETD3	84193	ENST00000331768	NM_032233.2
SETD4	54093	ENST00000332131	NM_017438.4
SETD5	55209	ENST00000402198	NM_001080517.2

SETD6	79918	ENST00000219315	NM_001160305.1
SETD7	80854	ENST00000274031	NM_001306199.1
SETDB1	9869	ENST00000271640	NM_001145415.1
SETDB2	83852	ENST00000354234	NM_031915.2
SF3B1	23451	ENST00000335508	NM_012433.2
SFPQ	6421		
SFRP1	6422	ENST00000220772	NM_003012.4
SFRP2	6423	ENST00000274063	NM_003013.2
SFRP4	6424		
SGK1	6446	ENST00000237305	NM_005627.3
SH2B3	10019	ENST00000341259	NM_005475.2
SH2D1A	4068	ENST00000371139	NM_002351.4
SH3GL1	6455		
SHOC2	8036	ENST00000369452	NM_007373.3
SHQ1	55164	ENST00000325599	NM_018130.2
SIX1	6495		
SLC1A2	6506		
SLC34A2	10568		
SLC45A3	85414		
SLFN11	91607	ENST00000308377	NM_001104587.1
SLX4	84464	ENST00000294008	NM_032444.2
SMAD2	4087	ENST00000262160	NM_001003652.3
SMAD3	4088	ENST00000327367	NM_005902.3
SMAD4	4089	ENST00000342988	NM_005359.5
SMARCA1	6594		
SMARCA2	6595	ENST00000349721	NM_001289396.1
SMARCA4	6597	ENST00000344626	NM_003072.3
SMARCB1	6598	ENST00000263121	NM_003073.3
SMARCD1	6602	ENST00000394963	NM_003076.4
SMARCE1	6605	ENST00000348513	NM_003079.4
SMC1A	8243	ENST00000322213	NM_006306.3
SMC3	9126	ENST00000361804	NM_005445.3
SMG1	23049	ENST00000446231	NM_015092.4
SMO	6608	ENST00000249373	NM_005631.4
SMYD3	64754	ENST00000388985	NM_001167740.1
SNCAIP	9627		
SND1	27044		
SNX29	92017		
SOCS1	8651	ENST00000332029	NM_003745.1
SOCS2	8835		
SOCS3	9021	ENST00000330871	NM_003955.4
SOS1	6654	ENST00000402219	NM_005633.3
SOX10	6663		
SOX17	64321	ENST00000297316	NM_022454.3
SOX2	6657	ENST00000325404	NM_003106.3
SOX9	6662	ENST00000245479	NM_000346.3
SP140	11262	ENST00000392045	NM_007237.4
SPEN	23013	ENST00000375759	NM_015001.2
SPOP	8405	ENST00000347630	NM_001007228.1
SPRED1	161742	ENST00000299084	NM_152594.2
SPRTN	83932	ENST00000295050	NM_032018.6
SRC	6714	ENST00000358208	NM_198291.2
SRSF2	6427	ENST00000359995	NM_003016.4
SRSF3	6428		

SS18	6760	ENST00000415083	NM_001007559.2
SS18L1	26039		
SSX1	6756		
SSX2	6757		
SSX4	6759		
STAG1	10274	ENST00000383202	NM_005862.2
STAG2	10735	ENST00000218089	NM_001042749.1
STAT1	6772	ENST00000361099	NM_007315.3
STAT2	6773	ENST00000314128	NM_005419.3
STAT3	6774	ENST00000264657	NM_139276.2
STAT4	6775		
STAT5A	6776	ENST00000345506	NM_003152.3
STAT5B	6777	ENST00000293328	NM_012448.3
STAT6	6778	ENST00000300134	NM_001178078.1
STIL	6491		
STK11	6794	ENST00000326873	NM_000455.4
STK19	8859	ENST00000375331	NM_004197.1
STK40	83931	ENST00000373129	NM_032017.1
STRN	6801		
SUFU	51684	ENST00000369902	NM_016169.3
SUZ12	23512	ENST00000322652	NM_015355.2
SYK	6850	ENST00000375746	NM_003177.5
TAF1	6872		
TAF15	8148		
TAL1	6886	ENST00000294339	NM_001287347.2
TAL2	6887		
TAP1	6890	ENST00000354258	NM_000593.5
TAP2	6891	ENST00000374899	NM_018833.2
TBL1XR1	79718	ENST00000430069	NM_024665.4
TBX3	6926	ENST00000257566	NM_016569.3
TCEA1	6917		
TCEB1	6921	ENST00000284811	NM_005648.3
TCF12	6938		
TCF3	6929	ENST00000344749	NM_001136139.2
TCF7L2	6934	ENST00000543371	NM_001146274.1
TCL1A	8115	ENST00000402399	NM_001098725.1
TCL1B	9623	ENST00000340722	NM_004918.3
TEC	7006		
TEK	7010	ENST00000380036	NM_000459.3
TERC	7012		
TERT	7015	ENST00000310581	NM_198253.2
TET1	80312	ENST00000373644	NM_030625.2
TET2	54790	ENST00000380013	NM_001127208.2
TET3	200424	ENST00000409262	NM_144993
TFE3	7030	ENST00000315869	NM_006521.5
TFEB	7942		
TFG	10342		
TFPT	29844		
TFRC	7037		
TGFBR1	7046	ENST00000374994	NM_004612.2
TGFBR2	7048	ENST00000359013	NM_001024847.2
TIPARP	25976		
TLE1	7088	ENST00000376499	NM_001303103.1
TLE2	7089	ENST00000262953	NM_003260.4

TLE3	7090	ENST00000558939	NM_005078.3
TLE4	7091	ENST00000376552	NM_007005.4
TLL2	7093		
TLX1	3195	ENST00000370196	NM_005521.3
TLX3	30012	ENST00000296921	NM_021025.2
TMEM127	55654	ENST00000258439	NM_001193304.2
TMEM30A	55754		
TMPRSS2	7113	ENST00000398585	NM_001135099.1
TMSB4XP8	7117		
TNFAIP3	7128	ENST00000237289	NM_006290.3
TNFRSF11A	8792		
TNFRSF14	8764	ENST00000355716	NM_003820.2
TNFRSF17	608		
TOP1	7150	ENST00000361337	NM_003286.2
TP53	7157	ENST00000269305	NM_000546.5
TP53BP1	7158	ENST00000382044	NM_001141980.1
TP63	8626	ENST00000264731	NM_003722.4
TPM3	7170		
TPM4	7171		
TPR	7175		
TRA	6955		
TRAF2	7186	ENST00000247668	NM_021138.3
TRAF3	7187	ENST00000392745	NM_003300.3
TRAF5	7188	ENST00000261464	NM_001033910.2
TRAF7	84231	ENST00000326181	NM_032271.2
TRB	6957		
TRD	6964		
TRG	6965		
TRIM24	8805		
TRIM27	5987		
TRIM33	51592		
TRIP11	9321		
TRIP13	9319	ENST00000166345	NM_004237.3
TRRAP	8295		
TSC1	7248	ENST00000298552	NM_000368.4
TSC2	7249	ENST00000219476	NM_000548.3
TSHR	7253	ENST00000298171	NM_000369.2
TTL	150465		
TUSC3	7991		
TYK2	7297	ENST00000264818	NM_003331.4
TYRO3	7301		
U2AF1	7307	ENST00000291552	NM_006758.2
U2AF2	11338	ENST00000308924	NM_007279.2
UBR5	51366	ENST00000520539	NM_015902.5
UPF1	5976	ENST00000262803	NM_002911.3
USP6	9098		
USP8	9101	ENST00000307179	NM_001128610.2
VAV1	7409	ENST00000602142	NM_005428.3
VAV2	7410	ENST00000371850	NM_001134398.1
VEGFA	7422	ENST00000523873	NM_001171623.1
VHL	7428	ENST00000256474	NM_000551.3
VTCN1	79679	ENST00000369458	NM_024626.3
WAS	7454		
WDR90	197335		

WHSC1	7468	ENST00000382891	NM_001042424.2
WHSC1L1	54904	ENST00000317025	NM_023034.1
WIF1	11197	ENST00000286574	NM_007191.4
WRN	7486		
WT1	7490	ENST00000332351	NM_024426.4
WWTR1	25937	ENST00000360632	NM_001168280.1
XBP1	7494	ENST00000216037	NM_005080.3
XIAP	331	ENST00000355640	NM_001167.3
XPA	7507		
XPC	7508		
XPO1	7514	ENST00000401558	NM_003400.3
XRCC2	7516	ENST00000359321	NM_005431.1
YAP1	10413	ENST00000282441	NM_001130145.2
YES1	7525	ENST00000314574	NM_005433.3
YPEL5	51646		
YWHAE	7531		
YY1	7528	ENST00000262238	NM_003403.4
YY1AP1	55249		
ZBTB16	7704		
ZBTB20	26137	ENST00000474710	NM_001164342.2
ZBTB7A	51341		
ZFHX3	463	ENST00000268489	NM_006885.3
ZFP36L1	677	ENST00000336440	NM_001244698.1
ZMYM2	7750		
ZMYM3	9203		
ZNF217	7764		
ZNF24	7572		
ZNF278	23598		
ZNF331	55422		
ZNF384	171017		
ZNF521	25925		
ZNF703	80139		
ZNRF3	84133	ENST00000544604	NM_001206998.1
ZRSR2	8233	ENST00000307771	NM_005089.3

# of occurrence within resources (Column D-J)	OncoKB Annotated	Is Oncogene	Is Tumor Suppressor Gene
2	No	No	No
7	Yes	Yes	No
3	Yes	Yes	No
1	No	No	No
1	No	No	No
1	No	No	No
1	No	No	No
2	Yes	No	Yes
4	Yes	Yes	No
2	No	No	No
1	No	No	No
1	No	No	No
2	No	No	No
2	No	No	No
1	No	No	No
2	No	No	No
2	Yes	Yes	No
3	Yes	No	No
1	Yes	No	Yes
7	Yes	Yes	No
6	Yes	Yes	No
5	Yes	Yes	No
2	Yes	No	No
7	Yes	Yes	No
4	Yes	Yes	Yes
7	Yes	No	Yes
3	Yes	No	Yes
7	Yes	No	Yes
1	No	No	No
2	Yes	Yes	Yes
1	No	No	No
7	Yes	Yes	No
5	Yes	Yes	No
2	No	No	No
2	No	No	No
2	Yes	Yes	Yes
2	No	No	No
2	Yes	Yes	No
7	Yes	No	Yes
5	Yes	No	Yes
6	Yes	No	Yes
2	Yes	No	Yes
2	Yes	Yes	No
2	Yes	No	No
2	Yes	No	Yes
2	Yes	No	Yes
2	Yes	No	No
3	Yes	No	Yes
2	No	No	No
1	No	No	No
1	No	No	No
7	Yes	No	Yes
3	Yes	No	Yes

3	Yes	Yes	No
1	No	No	No
2	No	No	No
7	Yes	No	Yes
1	No	No	No
1	No	No	No
2	Yes	No	No
2	Yes	No	Yes
6	Yes	No	Yes
7	Yes	No	Yes
2	Yes	No	Yes
2	Yes	Yes	No
5	Yes	Yes	No
5	Yes	Yes	No
7	Yes	No	Yes
4	Yes	No	Yes
5	Yes	Yes	No
6	Yes	No	Yes
3	Yes	No	No
2	Yes	No	Yes
7	Yes	No	Yes
6	Yes	No	Yes
1	No	No	No
3	Yes	No	Yes
5	Yes	No	Yes
2	No	No	No
4	Yes	No	Yes
7	Yes	Yes	No
4	Yes	No	No
3	Yes	No	Yes
2	No	No	No
2	No	No	No
6	Yes	Yes	No
2	No	No	No
3	Yes	Yes	No
1	No	No	No
7	Yes	No	Yes
5	Yes	No	Yes
5	Yes	Yes	No
5	Yes	No	No
5	Yes	No	Yes
4	Yes	No	Yes
7	Yes	Yes	No
7	Yes	No	Yes
7	Yes	No	Yes
1	No	No	No
6	Yes	Yes	No
6	Yes	No	Yes
1	No	No	No
5	Yes	No	Yes
2	No	No	No
6	Yes	Yes	No
1	No	No	No
1	No	No	No

1	No	No	No
1	No	No	No
1	No	No	No
6	Yes	Yes	No
2	No	No	No
1	No	No	No
7	Yes	Yes	No
3	Yes	No	No
2	No	No	No
1	No	No	No
6	Yes	No	Yes
2	No	No	No
6	Yes	No	Yes
7	Yes	No	Yes
1	No	No	No
1	No	No	No
1	No	No	No
1	No	No	No
1	No	No	No
1	Yes	Yes	No
6	Yes	Yes	No
6	Yes	Yes	No
6	Yes	Yes	No
6	Yes	Yes	No
1	No	No	No
2	No	No	No
6	Yes	Yes	No
3	Yes	No	No
2	Yes	Yes	No
1	No	No	No
3	Yes	No	Yes
2	No	No	No
2	No	No	No
6	Yes	Yes	No
6	Yes	Yes	No
3	Yes	Yes	No
7	Yes	No	Yes
7	Yes	No	Yes
1	No	No	No
6	Yes	No	Yes
6	Yes	Yes	No
6	Yes	Yes	No
5	Yes	Yes	No
4	Yes	No	Yes
6	Yes	No	Yes
7	Yes	No	Yes
5	Yes	No	Yes
6	Yes	No	Yes
2	No	No	No
7	Yes	No	Yes
3	Yes	No	No
1	No	No	No
1	No	No	No
1	No	No	No

5	Yes	No	Yes
6	Yes	No	Yes
1	No	No	No
1	No	No	No
7	Yes	No	Yes
4	Yes	No	Yes
1	No	No	No
1	No	No	No
1	No	No	No
2	No	No	No
2	No	No	No
2	Yes	No	Yes
1	No	No	No
1	No	No	No
2	No	No	No
2	No	No	No
1	No	No	No
1	No	No	No
2	Yes	No	Yes
2	Yes	Yes	No
2	No	No	No
2	No	No	No
7	Yes	No	Yes
5	Yes	Yes	No
6	Yes	Yes	No
1	No	No	No
1	No	No	No
3	Yes	No	No
1	No	No	No
6	Yes	No	No
6	Yes	Yes	No
6	Yes	No	Yes
3	Yes	Yes	No
2	No	No	No
7	Yes	Yes	No
2	Yes	No	Yes
4	Yes	No	No
1	No	No	No
4	Yes	No	Yes
6	Yes	Yes	No
2	Yes	Yes	No
5	Yes	No	Yes
1	No	No	No
2	Yes	Yes	No
3	Yes	No	No
7	Yes	No	Yes
1	No	No	No
3	Yes	No	No
1	No	No	No
3	Yes	No	No
1	No	No	No
6	Yes	Yes	No
2	No	No	No
4	Yes	No	Yes

1	Yes	Yes	No
1	Yes	No	Yes
1	No	No	No
2	No	No	No
3	Yes	Yes	No
4	Yes	No	Yes
4	Yes	No	Yes
1	Yes	No	No
1	Yes	No	No
1	Yes	No	No
1	Yes	No	No
4	Yes	No	No
2	No	No	No
4	Yes	Yes	No
7	Yes	No	Yes
3	Yes	No	Yes
5	Yes	Yes	No
4	Yes	No	No
3	Yes	No	Yes
1	No	No	No
3	Yes	No	Yes
3	Yes	No	Yes
1	No	No	No
3	Yes	Yes	No
2	No	No	No
2	Yes	No	Yes
5	Yes	No	Yes
3	Yes	Yes	No
7	Yes	Yes	No
2	Yes	No	Yes
3	Yes	No	No
1	No	No	No
5	Yes	Yes	No
3	Yes	Yes	No
3	Yes	Yes	Yes
2	No	No	No
1	No	No	No
2	No	No	No
1	Yes	No	Yes
1	No	No	No
1	No	No	No
2	No	No	No
2	No	No	No
7	Yes	No	Yes
2	Yes	No	Yes
4	Yes	No	No
3	Yes	No	Yes
5	Yes	No	Yes
4	Yes	No	No
4	Yes	Yes	Yes
5	Yes	No	Yes
1	No	No	No
2	Yes	Yes	No
2	No	No	No

7	Yes	Yes	No
6	Yes	Yes	No
6	Yes	Yes	No
1	No	No	No
4	Yes	No	Yes
4	Yes	No	Yes
5	Yes	No	Yes
4	Yes	No	Yes
3	Yes	No	Yes
6	Yes	Yes	No
4	Yes	No	Yes
2	Yes	No	Yes
6	Yes	Yes	No
2	Yes	No	Yes
3	Yes	No	No
1	No	No	No
5	Yes	Yes	No
4	Yes	Yes	No
4	Yes	Yes	No
6	Yes	No	Yes
4	Yes	Yes	No
1	No	No	No
1	No	No	No
1	No	No	No
3	Yes	Yes	No
7	Yes	Yes	Yes
2	No	No	No
1	No	No	No
3	Yes	No	Yes
6	Yes	No	Yes
3	Yes	No	Yes
6	Yes	No	Yes
6	Yes	No	Yes
4	Yes	No	Yes
2	No	No	No
2	No	No	No
3	No	No	No
2	No	No	No
5	Yes	No	Yes
4	Yes	No	Yes
1	No	No	No
4	Yes	No	Yes
1	No	No	No
7	Yes	No	Yes
2	No	No	No
2	No	No	No
1	No	No	No
3	Yes	No	No
2	No	No	No
1	No	No	No
2	No	No	No
5	Yes	Yes	No
2	No	No	No
5	Yes	Yes	No

5	Yes	Yes	No
2	No	No	No
6	Yes	Yes	No
2	No	No	No
7	Yes	Yes	No
7	Yes	Yes	No
6	Yes	Yes	No
5	Yes	No	Yes
2	No	No	No
1	No	No	No
6	Yes	No	Yes
3	Yes	Yes	No
5	Yes	Yes	No
7	Yes	Yes	No
5	Yes	Yes	No
1	No	No	No
1	No	No	No
4	Yes	Yes	Yes
2	Yes	Yes	Yes
7	Yes	Yes	Yes
5	Yes	No	Yes
2	No	No	No
2	No	No	No
5	Yes	Yes	Yes
1	No	No	No
2	No	No	No
6	Yes	No	Yes
2	Yes	Yes	No
2	No	No	No
3	Yes	Yes	No
2	Yes	Yes	No
2	Yes	Yes	No
1	No	No	No
1	No	No	No
2	No	No	No
6	Yes	No	No
6	Yes	Yes	No
7	Yes	Yes	Yes
1	No	No	No
1	No	No	No
2	No	No	No
4	Yes	Yes	No
1	No	No	No
7	Yes	Yes	No
3	Yes	Yes	No
4	Yes	Yes	No
7	Yes	Yes	No
7	Yes	Yes	No
3	Yes	Yes	No
1	No	No	No
1	No	No	No
1	No	No	No
2	No	No	No
3	Yes	No	Yes

3	Yes	Yes	No
5	Yes	No	Yes
1	No	No	No
5	Yes	Yes	No
2	Yes	Yes	No
1	No	No	No
6	Yes	Yes	No
1	Yes	No	No
4	Yes	No	No
3	Yes	No	No
4	Yes	Yes	No
3	Yes	Yes	Yes
3	Yes	Yes	No
2	No	No	No
2	No	No	No
5	Yes	Yes	No
3	Yes	Yes	No
2	No	No	No
1	No	No	No
2	Yes	No	Yes
4	Yes	No	No
3	Yes	No	Yes
3	Yes	Yes	No
3	Yes	No	No
3	Yes	No	No
3	Yes	No	No
3	Yes	Yes	No
3	Yes	No	No
3	Yes	No	No
2	Yes	No	No
3	Yes	No	No
3	Yes	No	No
3	Yes	No	No
3	Yes	No	No
6	Yes	No	No
3	Yes	No	No
3	Yes	No	No
3	Yes	No	No
3	Yes	No	No
3	Yes	No	No
3	Yes	No	No
3	Yes	No	No
3	Yes	No	No
2	No	No	No
1	Yes	No	No
3	Yes	No	No
3	Yes	No	No
3	Yes	No	No
4	Yes	No	Yes
3	Yes	No	Yes
2	Yes	No	Yes
2	No	No	No
2	No	No	No
2	No	No	No

7	Yes	No	Yes
1	No	No	No
1	No	No	No
2	No	No	No
2	No	No	No
1	No	No	No
2	No	No	No
3	Yes	Yes	Yes
2	No	No	No
2	No	No	No
2	No	No	No
2	No	No	No
7	Yes	Yes	No
1	No	No	No
2	No	No	No
2	No	No	No
1	No	No	No
3	Yes	Yes	No
5	Yes	No	Yes
7	Yes	Yes	No
7	Yes	Yes	No
3	Yes	No	Yes
3	Yes	Yes	No
5	Yes	Yes	No
3	Yes	Yes	No
2	No	No	No
2	No	No	No
2	No	No	No
1	No	No	No
5	Yes	Yes	No
6	Yes	No	No
1	No	No	No
3	Yes	Yes	Yes
3	Yes	No	No
1	No	No	No
2	No	No	No
2	Yes	Yes	No
1	No	No	No
5	Yes	Yes	No
3	Yes	No	Yes
4	Yes	Yes	Yes
3	Yes	No	No
5	Yes	No	Yes
1	No	No	No
3	Yes	No	Yes
3	Yes	Yes	No
3	Yes	No	Yes
1	No	No	No
6	Yes	Yes	No
3	Yes	No	Yes
3	Yes	Yes	No
5	Yes	Yes	No
1	No	No	No
2	No	No	No

7	Yes	Yes	Yes
7	Yes	Yes	No
7	Yes	Yes	No
3	Yes	Yes	Yes
1	No	No	No
6	Yes	Yes	No
3	Yes	No	No
1	No	No	No
2	Yes	No	No
1	No	No	No
1	No	No	No
1	No	No	No
6	Yes	Yes	No
7	Yes	No	Yes
7	Yes	No	Yes
6	Yes	Yes	No
2	No	No	No
6	Yes	No	Yes
1	No	No	No
2	No	No	No
7	Yes	Yes	No
1	Yes	No	Yes
5	Yes	Yes	Yes
2	Yes	Yes	No
1	No	No	No
2	No	No	No
1	No	No	No
6	Yes	No	Yes
3	Yes	No	Yes
6	Yes	No	Yes
7	Yes	No	Yes
3	Yes	No	No
3	Yes	No	No
7	Yes	Yes	No
2	Yes	Yes	No
1	No	No	No
2	No	No	No
4	Yes	No	Yes
4	Yes	No	Yes
3	Yes	Yes	No
1	No	No	No
2	No	No	No
1	No	No	No
1	No	No	No
5	Yes	Yes	No
3	Yes	Yes	No
2	No	No	No
1	No	No	No
2	No	No	No
1	Yes	Yes	Yes
1	Yes	Yes	No
1	No	No	No
2	Yes	Yes	Yes
1	No	No	No

2	No	No	No
4	Yes	Yes	No
3	Yes	No	Yes
2	Yes	Yes	Yes
4	Yes	No	No
3	Yes	Yes	No
1	No	No	No
5	Yes	No	No
1	No	No	No
7	Yes	Yes	No
6	Yes	Yes	No
6	Yes	No	Yes
7	Yes	No	Yes
5	Yes	Yes	No
4	Yes	Yes	No
1	No	No	No
1	No	No	No
6	Yes	Yes	No
3	Yes	Yes	No
3	Yes	No	No
4	Yes	No	Yes
1	Yes	No	Yes
5	Yes	Yes	No
3	Yes	No	No
6	Yes	Yes	No
6	Yes	Yes	No
1	No	No	No
3	Yes	Yes	No
7	Yes	Yes	Yes
5	Yes	Yes	No
1	No	No	No
7	Yes	No	Yes
1	No	No	No
7	Yes	Yes	No
3	Yes	No	Yes
2	Yes	Yes	No
1	No	No	No
6	Yes	Yes	Yes
1	No	No	No
1	No	No	No
2	No	No	No
7	Yes	No	Yes
4	Yes	No	No
3	Yes	Yes	No
1	No	No	No
2	No	No	No
2	No	No	No
2	No	No	No
1	No	No	No
2	Yes	No	Yes
2	Yes	No	No
7	Yes	Yes	No
5	Yes	No	Yes
2	No	No	No

7	Yes	No	Yes
5	Yes	No	Yes
7	Yes	No	Yes
3	Yes	Yes	No
5	Yes	Yes	No
2	No	No	No
3	Yes	No	Yes
4	Yes	Yes	No
3	Yes	No	Yes
1	No	No	No
6	Yes	Yes	No
2	No	No	No
6	Yes	No	Yes
3	No	No	No
6	Yes	Yes	No
6	Yes	Yes	No
6	Yes	Yes	No
7	Yes	Yes	No
2	No	No	No
2	No	No	No
1	No	No	No
1	No	No	No
4	Yes	No	No
1	No	No	No
1	No	No	No
2	Yes	Yes	No
1	No	No	No
5	Yes	No	Yes
1	No	No	No
2	No	No	No
3	Yes	Yes	No
1	No	No	No
5	Yes	No	Yes
4	Yes	No	No
3	Yes	Yes	No
2	No	No	No
3	Yes	No	No
7	Yes	No	Yes
7	Yes	No	Yes
1	No	No	No
2	Yes	Yes	No
7	Yes	Yes	No
1	No	No	No
2	No	No	No
5	Yes	No	Yes
1	No	No	No
2	No	No	No
6	Yes	Yes	No
3	Yes	No	Yes
1	No	No	No
1	No	No	No
7	Yes	Yes	Yes
7	Yes	Yes	Yes
4	Yes	Yes	Yes

3	Yes	Yes	Yes
7	Yes	No	Yes
3	Yes	Yes	No
7	Yes	Yes	No
2	Yes	Yes	No
5	Yes	Yes	Yes
5	Yes	Yes	No
3	Yes	No	Yes
6	Yes	Yes	No
5	Yes	Yes	No
6	Yes	Yes	No
3	Yes	Yes	No
2	No	No	No
2	No	No	No
4	Yes	No	No
3	Yes	Yes	No
3	Yes	No	No
2	No	No	No
1	No	No	No
1	No	No	No
1	No	No	No
5	Yes	No	Yes
2	No	No	No
1	No	No	No
3	Yes	Yes	No
1	No	No	No
3	Yes	Yes	No
6	Yes	No	Yes
4	Yes	No	Yes
4	Yes	No	Yes
1	No	No	No
1	No	No	No
1	No	No	No
2	No	No	No
7	Yes	No	Yes
2	No	No	No
2	Yes	Yes	No
7	Yes	No	Yes
2	No	No	No
1	No	No	No
3	Yes	No	No
1	No	No	No
2	No	No	No
1	No	No	No
5	Yes	Yes	No
1	No	No	No
6	Yes	Yes	No
2	No	No	No
3	Yes	Yes	No
7	Yes	Yes	No
6	Yes	Yes	No
2	No	No	No
3	Yes	No	No
2	Yes	No	Yes

2	No	No	No
2	Yes	Yes	No
3	Yes	Yes	No
1	No	No	No
6	Yes	No	Yes
4	Yes	No	Yes
2	No	No	No
2	Yes	No	Yes
1	No	No	No
4	Yes	No	No
3	Yes	No	No
7	Yes	Yes	No
5	Yes	Yes	No
3	Yes	Yes	No
4	Yes	Yes	No
7	Yes	No	Yes
4	Yes	No	Yes
3	Yes	No	Yes
6	Yes	No	No
2	No	No	No
3	Yes	Yes	No
4	Yes	Yes	No
3	Yes	No	No
3	Yes	No	Yes
2	No	No	No
3	Yes	No	Yes
5	Yes	No	Yes
3	Yes	No	No
5	Yes	No	Yes
5	Yes	No	Yes
1	No	No	No
5	Yes	No	Yes
2	No	No	No
1	No	No	No
5	Yes	Yes	No
1	No	No	No
4	Yes	Yes	No
1	No	No	No
7	Yes	No	Yes
1	No	No	No
3	Yes	No	No
4	Yes	No	Yes
1	No	No	No
7	Yes	No	Yes
3	Yes	No	No
2	No	No	No
4	Yes	No	No
1	No	No	No
2	Yes	Yes	No
6	Yes	No	No
4	Yes	Yes	No
3	Yes	No	No
1	No	No	No
2	No	No	No

1	Yes	No	No
1	No	No	No
2	No	No	No
7	Yes	No	Yes
7	Yes	No	Yes
1	No	No	No
1	No	No	No
3	Yes	No	No
2	Yes	Yes	Yes
7	Yes	Yes	No
1	No	No	No
3	Yes	No	Yes
1	No	No	No
1	No	No	No
1	No	No	No
3	Yes	No	Yes
1	No	No	No
2	No	No	No
3	Yes	No	Yes
4	Yes	No	Yes
2	No	No	No
3	Yes	Yes	No
2	No	No	No
5	Yes	Yes	No
3	Yes	Yes	No
6	Yes	No	Yes
4	Yes	No	Yes
5	Yes	No	Yes
5	Yes	No	Yes
4	Yes	No	Yes
4	Yes	No	Yes
4	Yes	No	No
4	Yes	No	No
6	Yes	Yes	No
1	No	No	No
1	No	No	No
2	No	No	No
6	Yes	No	No
3	Yes	No	Yes
1	No	No	No
7	Yes	No	Yes
5	Yes	No	Yes
3	Yes	Yes	Yes
3	Yes	No	Yes
4	Yes	No	Yes
5	Yes	Yes	No
2	Yes	No	No
2	Yes	No	Yes
7	Yes	Yes	No
3	Yes	Yes	No
3	Yes	Yes	No
5	Yes	Yes	No
2	No	No	No
5	Yes	Yes	No

3	Yes	Yes	No
1	No	No	No
2	No	No	No
1	No	No	No
7	Yes	No	Yes
2	Yes	No	Yes
6	Yes	Yes	No
1	No	No	No
2	No	No	No
1	No	No	No
2	No	No	No
3	Yes	Yes	No
3	Yes	Yes	No
5	Yes	Yes	No
3	Yes	Yes	No
3	Yes	Yes	No
3	Yes	Yes	No
2	No	No	No
1	No	No	No
3	Yes	No	Yes
6	Yes	No	Yes
4	Yes	Yes	No
1	No	No	No
3	Yes	No	No
3	Yes	No	Yes
1	No	No	No
1	No	No	No
2	Yes	No	Yes
1	No	No	No
2	Yes	Yes	No
2	No	No	No
6	Yes	No	Yes
4	Yes	No	Yes
6	Yes	No	Yes
6	Yes	No	Yes
6	Yes	No	Yes
1	No	No	No
1	No	No	No
1	No	No	No
1	No	No	No
1	No	No	No
2	Yes	Yes	Yes
2	Yes	No	No
3	Yes	No	Yes
3	Yes	No	Yes
3	Yes	No	Yes
2	No	No	No
5	Yes	Yes	No
2	Yes	Yes	No
2	Yes	No	No
7	Yes	No	Yes
2	Yes	No	No
2	Yes	No	No
2	Yes	No	No

2	Yes	No	No
2	Yes	No	No
3	Yes	Yes	Yes
2	Yes	No	Yes
7	Yes	Yes	No
1	No	No	No
1	Yes	No	Yes
1	Yes	Yes	Yes
1	No	No	No
4	Yes	Yes	No
4	Yes	No	Yes
3	Yes	No	Yes
2	No	No	No
3	Yes	Yes	No
3	Yes	No	Yes
1	No	No	No
1	No	No	No
2	No	No	No
1	No	No	No
2	Yes	No	Yes
3	Yes	No	Yes
7	Yes	No	Yes
4	Yes	No	Yes
7	Yes	No	Yes
1	No	No	No
2	Yes	No	Yes
7	Yes	No	Yes
7	Yes	No	Yes
4	Yes	No	No
3	Yes	Yes	Yes
3	Yes	No	Yes
3	Yes	No	Yes
2	Yes	No	Yes
7	Yes	Yes	No
3	Yes	Yes	No
1	No	No	No
1	No	No	No
1	No	No	No
7	Yes	No	Yes
1	No	No	No
2	Yes	No	Yes
3	Yes	Yes	No
1	No	No	No
3	Yes	No	Yes
6	Yes	Yes	No
5	Yes	Yes	Yes
2	Yes	No	Yes
6	Yes	No	Yes
7	Yes	No	Yes
3	Yes	No	Yes
2	Yes	No	Yes
6	Yes	Yes	No
6	Yes	No	No
2	No	No	No

3	Yes	Yes	No
1	No	No	No
2	No	No	No
2	No	No	No
2	No	No	No
2	Yes	No	Yes
7	Yes	No	Yes
1	Yes	No	No
1	Yes	No	No
6	Yes	Yes	No
1	No	No	No
4	Yes	Yes	No
5	Yes	Yes	No
4	Yes	Yes	No
1	No	No	No
7	Yes	No	Yes
3	Yes	Yes	No
3	Yes	No	No
1	No	No	No
6	Yes	No	Yes
5	Yes	No	Yes
6	Yes	Yes	No
1	No	No	No
2	No	No	No
3	Yes	Yes	No
2	No	No	No
3	Yes	No	No
3	Yes	No	No
4	Yes	No	Yes
5	Yes	No	Yes
1	No	No	No
3	Yes	No	No
1	No	No	No
5	Yes	No	Yes
4	Yes	No	Yes
3	Yes	Yes	No
1	Yes	Yes	No
1	No	No	No
4	Yes	No	No
1	No	No	No
5	Yes	Yes	No
5	Yes	No	Yes
7	Yes	No	Yes
2	Yes	No	Yes
3	Yes	Yes	No
1	No	No	No
2	No	No	No
1	No	No	No
1	No	No	No
3	Yes	No	Yes
6	Yes	No	Yes
1	No	No	No
1	Yes	No	No
1	Yes	No	No

1	Yes	No	No
1	Yes	No	No
1	No	No	No
3	Yes	Yes	No
3	Yes	Yes	No
4	Yes	No	Yes
1	No	No	No
6	Yes	No	No
1	No	No	No
7	Yes	No	Yes
1	No	No	No
6	Yes	No	Yes
2	No	No	No
5	Yes	No	Yes
7	Yes	No	Yes
3	Yes	No	Yes
5	Yes	Yes	Yes
2	No	No	No
2	No	No	No
1	No	No	No
2	Yes	Yes	No
4	Yes	No	No
3	Yes	No	Yes
3	Yes	No	Yes
5	Yes	No	No
2	Yes	Yes	No
2	Yes	Yes	No
2	Yes	Yes	No
2	No	No	No
1	No	No	No
1	No	No	No
2	No	No	No
2	Yes	Yes	Yes
1	No	No	No
7	Yes	No	Yes
6	Yes	No	Yes
6	Yes	Yes	No
1	No	No	No
1	No	No	No
3	Yes	Yes	No
1	No	No	No
7	Yes	Yes	No
3	Yes	No	No
3	Yes	Yes	No
3	Yes	No	No
2	No	No	No
3	Yes	Yes	No
2	Yes	Yes	No
2	Yes	Yes	No
4	Yes	Yes	No
7	Yes	No	Yes
3	Yes	No	No
1	No	No	No
1	No	No	No

6	Yes	Yes	No
6	Yes	No	No
2	Yes	No	Yes
1	No	No	No
7	Yes	Yes	Yes
4	Yes	Yes	No
3	Yes	Yes	No
3	Yes	Yes	No
1	No	No	No
1	No	No	No
6	Yes	Yes	No
4	Yes	No	Yes
3	Yes	Yes	No
3	Yes	Yes	No
1	No	No	No
1	No	No	No
1	Yes	Yes	No
1	No	No	No
2	No	No	No
1	Yes	Yes	Yes
1	No	No	No
4	Yes	No	Yes
1	Yes	No	Yes
2	No	No	No
1	No	No	No
2	No	No	No
1	No	No	No
1	No	No	No
1	No	No	No
2	No	No	No
2	No	No	No
2	No	No	No
2	Yes	No	Yes
5	Yes	No	No

MSK-IMPACT	MSK-HEME	FOUNDATION ONE	FOUNDATION ONE HEME	Vogelstein
No	No	No	Yes	No
Yes	Yes	Yes	Yes	Yes
No	No	No	Yes	No
No	No	No	No	No
No	No	No	No	No
No	No	No	Yes	No
No	No	No	Yes	No
No	Yes	No	No	No
Yes	Yes	No	No	No
No	No	Yes	No	Yes
No	No	No	No	No
No	No	No	Yes	No
No	No	No	Yes	No
No	No	No	Yes	No
No	No	No	No	No
No	No	No	Yes	No
Yes	No	No	No	No
Yes	Yes	No	No	No
No	No	No	No	No
Yes	Yes	Yes	Yes	Yes
Yes	Yes	Yes	Yes	No
Yes	Yes	Yes	Yes	No
Yes	No	No	No	No
Yes	Yes	Yes	Yes	Yes
Yes	Yes	Yes	No	No
Yes	Yes	Yes	Yes	Yes
Yes	Yes	No	No	No
Yes	Yes	Yes	Yes	Yes
No	No	No	Yes	No
Yes	No	No	No	No
No	No	No	No	No
Yes	Yes	Yes	Yes	Yes
Yes	Yes	Yes	Yes	No
No	No	Yes	Yes	No
No	No	No	Yes	No
Yes	No	No	No	No
No	No	No	Yes	No
No	Yes	No	No	No
Yes	Yes	Yes	Yes	Yes
Yes	Yes	No	No	Yes
Yes	Yes	No	Yes	Yes
No	Yes	No	No	No
No	Yes	No	No	No
No	Yes	No	No	No
No	Yes	No	No	No
No	Yes	No	No	No
No	Yes	No	No	No
Yes	Yes	No	No	No
No	No	No	Yes	No
No	No	No	Yes	No
No	No	No	No	No
Yes	Yes	Yes	Yes	Yes
Yes	Yes	No	No	No

No	No	No	Yes	No
No	No	No	Yes	No
No	No	No	Yes	No
Yes	Yes	Yes	Yes	Yes
No	No	No	No	No
No	No	No	No	No
No	Yes	No	No	No
No	Yes	No	No	No
Yes	Yes	Yes	Yes	No
Yes	Yes	Yes	Yes	Yes
No	Yes	No	No	No
Yes	No	No	No	No
Yes	Yes	Yes	Yes	No
Yes	Yes	Yes	Yes	No
Yes	Yes	Yes	Yes	Yes
Yes	Yes	No	No	No
Yes	Yes	Yes	Yes	No
Yes	Yes	No	Yes	Yes
Yes	Yes	No	No	No
No	Yes	No	No	No
Yes	Yes	Yes	Yes	Yes
Yes	Yes	Yes	Yes	No
No	No	No	No	No
Yes	Yes	No	No	No
Yes	Yes	No	Yes	No
No	No	No	Yes	No
No	Yes	No	Yes	No
Yes	Yes	Yes	Yes	Yes
Yes	Yes	Yes	No	No
Yes	Yes	No	No	No
No	No	Yes	Yes	No
No	No	No	Yes	No
Yes	Yes	Yes	Yes	No
No	No	No	Yes	No
No	No	No	Yes	No
No	No	No	No	No
Yes	Yes	Yes	Yes	Yes
No	Yes	Yes	Yes	No
No	Yes	Yes	Yes	No
Yes	Yes	No	Yes	No
Yes	Yes	No	Yes	No
Yes	Yes	No	No	No
Yes	Yes	Yes	Yes	Yes
Yes	Yes	Yes	Yes	Yes
Yes	Yes	Yes	Yes	Yes
No	No	No	No	No
Yes	Yes	Yes	Yes	No
Yes	Yes	Yes	Yes	No
No	No	No	Yes	No
No	Yes	Yes	Yes	No
No	No	Yes	Yes	No
Yes	Yes	Yes	Yes	No
No	No	No	Yes	No
No	No	No	No	No

No	No	No	No	No
No	No	No	No	No
No	No	No	Yes	No
Yes	Yes	Yes	Yes	No
No	No	No	Yes	No
No	No	No	No	No
Yes	Yes	Yes	Yes	Yes
Yes	Yes	No	No	No
No	No	No	Yes	No
No	No	No	No	No
Yes	Yes	Yes	No	Yes
No	No	No	Yes	No
Yes	Yes	Yes	Yes	No
Yes	Yes	Yes	Yes	Yes
No	No	No	No	No
No	No	No	No	No
No	No	No	No	No
No	No	No	Yes	No
No	No	No	No	No
No	No	No	No	No
Yes	Yes	Yes	Yes	No
Yes	Yes	Yes	Yes	No
Yes	Yes	Yes	Yes	No
Yes	Yes	Yes	Yes	No
No	No	No	Yes	No
No	No	Yes	Yes	No
Yes	Yes	Yes	Yes	No
Yes	Yes	No	No	No
No	Yes	No	No	No
No	No	No	Yes	No
No	Yes	No	Yes	No
No	No	Yes	Yes	No
No	No	Yes	No	No
Yes	Yes	Yes	Yes	No
Yes	Yes	Yes	Yes	No
Yes	Yes	No	No	No
Yes	Yes	Yes	Yes	Yes
Yes	Yes	Yes	Yes	Yes
No	No	No	No	No
Yes	Yes	Yes	Yes	No
Yes	Yes	Yes	Yes	No
Yes	Yes	Yes	Yes	No
Yes	Yes	Yes	Yes	No
Yes	Yes	Yes	No	No
Yes	Yes	Yes	Yes	No
Yes	Yes	Yes	Yes	Yes
Yes	Yes	Yes	Yes	No
Yes	Yes	Yes	Yes	No
No	No	No	Yes	No
Yes	Yes	Yes	Yes	Yes
Yes	Yes	No	No	No
No	No	No	No	No
No	No	No	Yes	No
No	No	No	No	No

Yes	Yes	Yes	Yes	No
Yes	Yes	Yes	Yes	No
No	No	No	Yes	No
No	No	No	Yes	No
Yes	Yes	Yes	Yes	Yes
No	Yes	No	Yes	No
No	No	No	Yes	No
No	No	No	No	No
No	No	No	Yes	No
No	No	No	Yes	No
No	No	No	Yes	No
Yes	No	No	No	No
No	No	No	No	No
No	No	No	No	No
No	No	No	Yes	No
No	No	No	Yes	No
No	No	No	No	No
No	No	No	Yes	No
No	Yes	No	No	No
No	No	No	No	No
No	No	No	Yes	No
No	No	No	Yes	No
Yes	Yes	Yes	Yes	Yes
Yes	Yes	Yes	Yes	No
Yes	Yes	No	Yes	Yes
No	No	No	No	No
No	No	No	No	No
Yes	Yes	No	No	No
No	No	No	Yes	No
Yes	Yes	Yes	Yes	Yes
Yes	Yes	Yes	Yes	No
Yes	Yes	Yes	Yes	No
Yes	Yes	No	No	No
No	No	Yes	Yes	No
Yes	Yes	Yes	Yes	Yes
Yes	No	No	No	No
Yes	Yes	Yes	No	No
No	No	Yes	No	No
No	Yes	No	Yes	No
Yes	Yes	Yes	Yes	No
Yes	No	No	No	No
Yes	Yes	No	No	Yes
No	No	Yes	No	No
Yes	No	No	No	No
Yes	Yes	No	No	No
Yes	Yes	Yes	Yes	Yes
No	No	No	No	No
Yes	Yes	No	No	No
No	No	No	No	No
No	No	No	Yes	No
No	No	Yes	No	No
Yes	Yes	Yes	Yes	No
No	No	No	Yes	No
No	Yes	No	Yes	No

No	No	No	No	No
No	No	No	No	No
No	No	No	No	No
No	No	No	Yes	No
No	No	No	Yes	No
Yes	Yes	No	No	No
Yes	Yes	Yes	No	No
No	No	No	No	No
No	No	No	No	No
No	No	No	No	No
No	No	No	No	No
Yes	Yes	No	No	No
No	No	No	Yes	No
Yes	Yes	No	No	Yes
Yes	Yes	Yes	Yes	Yes
Yes	Yes	No	No	No
Yes	Yes	Yes	Yes	No
Yes	Yes	No	No	No
No	Yes	No	Yes	No
No	No	No	Yes	No
No	Yes	No	Yes	No
Yes	Yes	No	No	No
No	No	No	Yes	No
Yes	Yes	No	No	No
No	No	No	Yes	No
No	No	No	Yes	No
Yes	Yes	Yes	Yes	No
Yes	Yes	No	No	No
Yes	Yes	Yes	Yes	Yes
No	Yes	No	No	No
Yes	Yes	No	No	No
No	No	No	No	No
Yes	Yes	No	Yes	No
Yes	Yes	No	No	No
Yes	Yes	No	No	No
No	No	No	Yes	No
No	No	No	No	No
No	No	No	Yes	No
No	No	No	No	No
No	No	No	Yes	No
No	No	No	Yes	No
No	No	No	Yes	No
No	No	Yes	Yes	No
Yes	Yes	Yes	Yes	Yes
No	Yes	No	No	No
Yes	Yes	No	No	No
Yes	Yes	No	No	No
Yes	Yes	Yes	Yes	No
Yes	Yes	No	Yes	No
Yes	Yes	No	Yes	No
Yes	Yes	Yes	Yes	No
No	No	Yes	No	No
No	No	No	Yes	No
No	No	No	Yes	No

Yes	Yes	Yes	Yes	Yes
Yes	Yes	Yes	Yes	No
Yes	Yes	Yes	Yes	No
No	No	No	No	No
Yes	Yes	No	No	No
Yes	Yes	No	No	No
Yes	Yes	Yes	No	No
Yes	Yes	No	No	No
Yes	Yes	No	No	No
Yes	Yes	Yes	Yes	No
Yes	Yes	Yes	No	No
No	Yes	No	No	No
Yes	Yes	Yes	Yes	No
Yes	No	No	No	No
No	Yes	No	No	No
No	No	No	Yes	No
Yes	Yes	No	Yes	No
No	No	Yes	Yes	No
No	No	Yes	Yes	No
Yes	Yes	Yes	Yes	No
No	No	Yes	Yes	No
No	No	No	Yes	No
No	No	No	No	No
No	No	No	No	No
Yes	Yes	No	No	No
Yes	Yes	Yes	Yes	Yes
No	No	Yes	No	No
No	No	No	Yes	No
Yes	Yes	No	No	No
Yes	Yes	Yes	Yes	No
Yes	Yes	No	No	No
Yes	Yes	Yes	Yes	No
Yes	Yes	Yes	Yes	No
No	Yes	No	Yes	No
No	No	No	Yes	No
No	No	No	Yes	No
No	No	Yes	Yes	No
No	No	Yes	Yes	No
No	Yes	Yes	Yes	No
Yes	Yes	No	No	No
No	No	No	No	No
No	Yes	No	Yes	No
No	No	No	Yes	No
Yes	Yes	Yes	Yes	Yes
No	No	No	Yes	No
No	No	No	Yes	No
No	No	No	No	No
No	No	No	Yes	No
No	No	Yes	Yes	No
No	No	Yes	No	No
No	No	Yes	Yes	No
Yes	Yes	Yes	Yes	No
No	No	Yes	Yes	No
Yes	Yes	Yes	Yes	No

Yes	Yes	Yes	Yes	No
No	No	Yes	Yes	No
Yes	Yes	Yes	Yes	No
No	No	No	Yes	No
Yes	Yes	Yes	Yes	Yes
Yes	Yes	Yes	Yes	Yes
Yes	Yes	Yes	Yes	No
Yes	Yes	Yes	No	No
No	No	No	Yes	No
No	No	No	No	No
Yes	Yes	Yes	Yes	No
No	No	No	Yes	No
Yes	Yes	Yes	Yes	No
Yes	Yes	Yes	Yes	Yes
Yes	Yes	No	Yes	No
No	No	No	Yes	No
No	No	No	Yes	No
Yes	Yes	No	No	No
Yes	No	No	No	No
Yes	Yes	Yes	Yes	Yes
Yes	Yes	No	Yes	No
No	No	No	Yes	No
No	No	No	Yes	No
Yes	Yes	No	Yes	No
No	No	No	Yes	No
No	No	No	Yes	No
Yes	Yes	Yes	No	Yes
No	Yes	No	No	No
No	No	No	Yes	No
Yes	Yes	No	No	No
Yes	No	No	No	No
Yes	No	No	No	No
No	No	Yes	No	No
No	No	No	Yes	No
No	No	No	Yes	No
Yes	Yes	No	Yes	Yes
Yes	Yes	No	Yes	Yes
Yes	Yes	Yes	Yes	Yes
No	No	Yes	No	No
No	No	Yes	No	No
No	No	Yes	Yes	No
Yes	Yes	No	Yes	No
No	No	No	Yes	No
Yes	Yes	Yes	Yes	Yes
No	Yes	No	Yes	No
No	Yes	Yes	Yes	No
Yes	Yes	Yes	Yes	Yes
Yes	Yes	Yes	Yes	Yes
Yes	Yes	No	No	No
No	No	No	No	No
No	No	No	No	No
No	No	No	No	No
No	No	No	Yes	No
Yes	Yes	No	No	No

Yes	Yes	No	No	No
Yes	Yes	No	Yes	No
No	No	Yes	No	No
Yes	Yes	Yes	Yes	No
No	Yes	No	No	No
No	No	No	Yes	No
Yes	Yes	Yes	No	Yes
No	No	No	No	No
Yes	Yes	No	No	No
Yes	Yes	No	No	No
No	Yes	Yes	Yes	No
No	Yes	No	Yes	No
No	Yes	No	Yes	No
No	No	No	Yes	No
No	No	No	Yes	No
Yes	Yes	Yes	Yes	No
No	Yes	No	No	No
No	No	No	Yes	No
No	Yes	No	No	No
No	Yes	No	No	No
Yes	Yes	No	Yes	No
No	Yes	No	Yes	No
No	Yes	No	Yes	No
No	Yes	No	Yes	No
No	Yes	No	Yes	No
No	Yes	No	Yes	No
No	Yes	No	Yes	No
No	Yes	No	Yes	No
Yes	Yes	No	No	No
No	Yes	No	No	No
No	Yes	No	Yes	No
No	Yes	No	Yes	No
No	Yes	No	Yes	No
Yes	Yes	No	No	No
Yes	Yes	No	Yes	Yes
Yes	Yes	No	No	No
Yes	Yes	No	No	No
Yes	Yes	No	No	No
Yes	Yes	No	No	No
Yes	Yes	No	No	No
Yes	Yes	No	No	No
Yes	Yes	No	No	No
Yes	Yes	No	No	No
No	No	No	Yes	No
No	No	No	No	No
Yes	Yes	No	No	No
Yes	Yes	No	No	No
Yes	Yes	No	No	No
Yes	Yes	No	No	No
Yes	Yes	No	No	No
Yes	No	No	No	No
No	No	No	Yes	No
No	No	No	Yes	No
No	No	No	Yes	No

Yes	Yes	Yes	Yes	Yes
No	No	No	No	No
No	No	No	No	No
No	No	No	Yes	No
No	No	No	Yes	No
No	No	No	Yes	No
No	No	No	Yes	No
Yes	Yes	No	No	No
No	No	No	Yes	No
No	No	No	Yes	No
No	No	No	Yes	No
No	No	No	Yes	No
Yes	Yes	Yes	Yes	Yes
No	No	Yes	No	No
No	No	No	Yes	No
No	No	No	Yes	No
No	No	No	Yes	No
Yes	Yes	No	No	No
Yes	Yes	Yes	Yes	No
Yes	Yes	Yes	Yes	Yes
Yes	Yes	Yes	Yes	Yes
Yes	Yes	No	No	No
Yes	Yes	No	No	No
Yes	Yes	Yes	Yes	No
Yes	Yes	No	No	No
No	No	No	Yes	No
No	No	No	Yes	No
No	No	No	Yes	No
No	No	No	No	No
Yes	Yes	Yes	Yes	No
Yes	Yes	Yes	Yes	No
No	No	No	Yes	No
No	Yes	No	Yes	No
Yes	Yes	No	No	No
No	No	No	No	No
No	No	No	Yes	No
No	No	No	Yes	No
No	No	No	No	No
Yes	Yes	No	Yes	No
Yes	Yes	No	No	No
Yes	Yes	No	Yes	No
Yes	Yes	No	No	No
Yes	Yes	Yes	Yes	No
No	No	No	Yes	No
Yes	Yes	No	No	No
Yes	Yes	No	No	No
No	Yes	No	Yes	No
No	No	Yes	No	No
Yes	Yes	Yes	Yes	No
No	Yes	No	Yes	No
Yes	Yes	No	No	No
Yes	Yes	Yes	Yes	No
No	No	No	No	No
No	No	No	Yes	No

Yes	Yes	Yes	Yes	Yes
Yes	Yes	Yes	Yes	Yes
Yes	Yes	Yes	Yes	Yes
No	Yes	No	Yes	No
No	No	No	Yes	No
Yes	Yes	Yes	Yes	No
No	No	No	Yes	No
No	No	No	No	No
Yes	No	No	No	No
No	No	No	No	No
No	No	No	Yes	No
No	No	No	Yes	No
Yes	Yes	Yes	Yes	No
Yes	Yes	Yes	Yes	Yes
Yes	Yes	Yes	Yes	Yes
Yes	Yes	Yes	Yes	No
No	No	No	Yes	No
Yes	Yes	Yes	Yes	No
No	No	Yes	No	No
No	No	No	Yes	No
Yes	Yes	Yes	Yes	Yes
No	No	No	No	No
Yes	Yes	No	No	Yes
Yes	No	No	No	No
No	No	No	No	No
No	No	Yes	Yes	No
No	No	No	No	No
Yes	Yes	Yes	Yes	No
Yes	Yes	No	No	No
Yes	Yes	No	Yes	Yes
Yes	Yes	Yes	Yes	Yes
Yes	Yes	No	No	No
Yes	Yes	No	No	No
Yes	Yes	Yes	Yes	Yes
No	Yes	No	No	No
No	No	No	No	No
No	No	No	Yes	No
Yes	Yes	No	No	No
Yes	Yes	No	No	No
No	Yes	No	No	No
No	No	No	Yes	No
No	No	No	Yes	No
No	No	No	No	No
No	No	No	No	No
Yes	Yes	No	Yes	No
No	No	No	Yes	No
No	No	No	Yes	No
No	No	No	No	No
No	No	No	Yes	No
No	No	No	No	No
No	No	No	No	No
No	No	No	Yes	No
No	Yes	No	No	No
No	No	Yes	No	No

No	No	No	Yes	No
Yes	Yes	Yes	No	No
Yes	No	No	No	No
Yes	No	No	No	No
No	No	Yes	Yes	No
No	No	No	Yes	No
No	No	No	Yes	No
Yes	Yes	No	Yes	No
No	No	No	No	No
Yes	Yes	Yes	Yes	Yes
Yes	Yes	Yes	Yes	No
Yes	Yes	Yes	Yes	No
Yes	Yes	Yes	Yes	Yes
Yes	Yes	Yes	No	No
Yes	Yes	No	Yes	No
No	No	No	Yes	No
No	No	No	Yes	No
Yes	Yes	Yes	Yes	No
Yes	Yes	No	No	No
Yes	Yes	No	No	No
Yes	Yes	No	No	No
No	No	No	No	No
Yes	Yes	Yes	Yes	No
Yes	Yes	No	No	No
Yes	Yes	Yes	Yes	No
Yes	Yes	Yes	Yes	No
No	No	No	Yes	No
No	No	No	Yes	No
Yes	Yes	Yes	Yes	Yes
Yes	Yes	Yes	Yes	No
No	No	No	Yes	No
Yes	Yes	Yes	Yes	Yes
No	No	Yes	No	No
Yes	Yes	Yes	Yes	Yes
Yes	Yes	No	No	No
No	Yes	No	No	No
No	No	No	Yes	No
Yes	Yes	Yes	Yes	No
No	No	No	Yes	No
No	No	Yes	No	No
No	No	No	Yes	No
Yes	Yes	Yes	Yes	Yes
Yes	No	No	Yes	No
No	No	No	Yes	No
No	No	No	No	No
No	No	No	Yes	No
No	No	No	Yes	No
No	No	No	Yes	No
No	No	No	Yes	No
No	Yes	No	No	No
No	Yes	No	No	No
Yes	Yes	Yes	Yes	Yes
Yes	Yes	Yes	Yes	No
No	No	No	Yes	No

Yes	Yes	Yes	Yes	Yes
Yes	Yes	Yes	Yes	No
Yes	Yes	Yes	Yes	Yes
Yes	Yes	No	No	No
Yes	Yes	No	Yes	No
No	No	No	Yes	No
Yes	Yes	No	No	No
Yes	Yes	Yes	No	No
Yes	No	Yes	No	No
No	No	No	No	No
Yes	Yes	Yes	Yes	No
No	No	No	Yes	No
Yes	Yes	Yes	Yes	No
No	No	Yes	Yes	No
Yes	Yes	Yes	Yes	No
Yes	Yes	Yes	Yes	No
Yes	Yes	Yes	Yes	No
Yes	Yes	Yes	Yes	Yes
No	No	No	Yes	No
No	No	No	Yes	No
No	No	No	Yes	No
No	No	No	No	No
Yes	Yes	No	No	No
No	No	No	No	No
No	No	No	Yes	No
Yes	No	No	No	No
No	No	No	Yes	No
Yes	Yes	Yes	No	No
No	No	No	No	No
No	No	No	Yes	No
Yes	Yes	No	No	No
No	No	No	No	No
Yes	Yes	No	No	Yes
No	Yes	No	Yes	No
No	Yes	No	Yes	No
No	No	No	Yes	No
Yes	Yes	No	No	No
Yes	Yes	Yes	Yes	Yes
Yes	Yes	Yes	Yes	Yes
No	No	No	No	No
No	Yes	No	No	No
Yes	Yes	Yes	Yes	Yes
No	No	No	No	No
No	No	No	Yes	No
Yes	Yes	Yes	Yes	No
No	No	No	No	No
No	No	No	Yes	No
Yes	Yes	Yes	Yes	No
Yes	Yes	No	No	No
No	No	No	Yes	No
No	No	No	No	No
Yes	Yes	Yes	Yes	Yes
Yes	Yes	Yes	Yes	Yes
Yes	Yes	Yes	No	No

Yes	Yes	No	No	No
Yes	Yes	Yes	Yes	Yes
No	No	No	Yes	No
Yes	Yes	Yes	Yes	Yes
No	No	No	No	No
Yes	Yes	No	Yes	No
No	Yes	Yes	Yes	No
Yes	Yes	No	No	No
Yes	Yes	Yes	Yes	No
Yes	Yes	Yes	Yes	No
Yes	Yes	Yes	Yes	No
Yes	Yes	No	No	No
No	No	No	Yes	No
No	No	No	Yes	No
Yes	Yes	No	Yes	No
No	No	No	Yes	No
No	No	Yes	No	No
No	No	No	Yes	No
No	No	No	No	No
No	No	No	No	No
No	No	No	Yes	No
No	Yes	Yes	Yes	No
No	No	No	Yes	No
No	No	No	Yes	No
Yes	Yes	No	No	No
No	No	No	Yes	No
Yes	Yes	No	No	No
Yes	Yes	Yes	Yes	No
Yes	Yes	Yes	No	No
Yes	Yes	Yes	No	No
No	No	Yes	No	No
No	No	Yes	No	No
No	No	No	Yes	No
No	No	No	Yes	No
Yes	Yes	Yes	Yes	Yes
No	No	No	Yes	No
No	No	No	No	No
Yes	Yes	Yes	Yes	Yes
No	No	No	Yes	No
No	No	No	Yes	No
No	Yes	No	Yes	No
No	No	No	Yes	No
No	No	No	Yes	No
No	No	No	Yes	No
Yes	Yes	Yes	Yes	No
No	No	No	Yes	No
Yes	Yes	Yes	Yes	No
No	No	No	Yes	No
No	No	No	Yes	No
Yes	Yes	Yes	Yes	Yes
Yes	Yes	Yes	Yes	No
No	No	Yes	Yes	No
Yes	Yes	No	No	No
No	Yes	No	No	No

No	No	No	Yes	No
Yes	No	No	No	No
Yes	Yes	No	No	No
No	No	No	Yes	No
Yes	Yes	No	Yes	Yes
Yes	Yes	No	No	No
No	No	No	Yes	No
No	Yes	No	No	No
No	No	Yes	No	No
Yes	Yes	Yes	No	No
Yes	Yes	No	No	No
Yes	Yes	Yes	Yes	Yes
Yes	Yes	Yes	No	No
Yes	Yes	No	No	No
Yes	Yes	No	Yes	No
Yes	Yes	Yes	Yes	Yes
Yes	Yes	No	Yes	No
Yes	Yes	No	No	No
Yes	Yes	Yes	Yes	No
No	No	No	Yes	No
No	Yes	No	No	No
Yes	Yes	No	Yes	No
Yes	Yes	No	No	No
Yes	Yes	No	No	No
No	No	No	Yes	No
Yes	Yes	No	No	No
Yes	Yes	Yes	No	No
Yes	Yes	No	No	No
Yes	Yes	Yes	No	No
Yes	Yes	Yes	No	No
No	No	No	No	No
Yes	Yes	No	Yes	No
No	No	No	Yes	No
No	No	No	No	No
Yes	Yes	Yes	No	No
No	No	No	No	No
Yes	Yes	No	No	No
No	No	No	Yes	No
Yes	Yes	Yes	Yes	Yes
No	No	Yes	No	No
Yes	Yes	No	No	No
Yes	Yes	No	No	No
No	No	No	No	No
Yes	Yes	Yes	Yes	Yes
Yes	Yes	No	No	No
No	No	No	Yes	No
Yes	Yes	No	No	No
No	No	No	No	No
No	No	No	No	No
Yes	Yes	Yes	Yes	No
Yes	Yes	Yes	No	No
Yes	Yes	No	No	No
No	No	No	Yes	No
No	No	No	Yes	No

No	No	No	No	No
No	No	No	Yes	No
No	No	No	Yes	No
Yes	Yes	Yes	Yes	Yes
Yes	Yes	Yes	Yes	Yes
No	No	No	No	No
No	No	No	Yes	No
Yes	Yes	No	No	No
No	Yes	No	No	No
Yes	Yes	Yes	Yes	Yes
No	No	No	No	No
No	Yes	No	Yes	No
No	No	No	Yes	No
No	No	No	No	No
No	No	No	No	No
Yes	Yes	No	No	No
No	No	No	No	No
No	No	Yes	Yes	No
Yes	Yes	No	No	No
Yes	Yes	No	No	No
No	No	Yes	No	No
Yes	Yes	No	No	No
No	No	No	Yes	No
Yes	Yes	Yes	No	No
Yes	Yes	No	No	No
Yes	Yes	Yes	Yes	No
Yes	Yes	No	Yes	No
Yes	Yes	Yes	Yes	No
Yes	Yes	Yes	No	No
Yes	Yes	Yes	No	No
Yes	Yes	Yes	No	No
Yes	Yes	Yes	No	No
Yes	Yes	Yes	No	No
Yes	Yes	Yes	Yes	No
No	No	No	Yes	No
No	No	No	No	No
No	No	No	Yes	No
Yes	Yes	Yes	Yes	No
Yes	Yes	No	No	No
No	No	No	Yes	No
Yes	Yes	Yes	Yes	Yes
Yes	Yes	Yes	No	No
No	No	No	Yes	No
Yes	Yes	No	No	No
Yes	Yes	No	No	No
Yes	Yes	Yes	No	No
No	No	No	Yes	No
Yes	No	No	No	No
Yes	Yes	Yes	Yes	Yes
Yes	Yes	No	No	No
Yes	Yes	No	No	No
Yes	Yes	No	Yes	No
No	No	No	Yes	No
Yes	Yes	Yes	Yes	No

Yes	Yes	No	No	No
No	No	No	No	No
No	No	No	Yes	No
No	No	No	Yes	No
Yes	Yes	Yes	Yes	Yes
No	Yes	No	No	No
Yes	Yes	Yes	Yes	No
No	No	No	No	No
No	No	No	Yes	No
No	No	No	No	No
No	No	No	Yes	No
Yes	Yes	No	No	No
Yes	Yes	No	No	No
Yes	Yes	Yes	Yes	No
Yes	Yes	No	No	No
Yes	Yes	No	No	No
Yes	Yes	No	No	No
No	No	Yes	No	No
No	No	No	No	No
Yes	Yes	No	No	No
Yes	Yes	No	Yes	Yes
No	Yes	No	Yes	No
No	No	No	Yes	No
Yes	Yes	No	No	No
Yes	Yes	No	No	No
No	No	No	Yes	No
No	No	No	No	No
No	Yes	No	No	No
No	No	No	No	No
Yes	No	No	No	No
No	No	Yes	No	No
Yes	Yes	Yes	Yes	No
Yes	Yes	No	No	No
Yes	Yes	Yes	Yes	No
Yes	Yes	Yes	Yes	No
Yes	Yes	Yes	Yes	No
No	No	No	Yes	No
No	No	No	Yes	No
No	No	No	Yes	No
No	No	No	Yes	No
No	No	No	Yes	No
Yes	No	No	No	No
Yes	No	No	No	No
Yes	Yes	No	No	No
Yes	Yes	No	No	No
Yes	Yes	No	No	No
No	No	No	Yes	No
No	Yes	No	Yes	Yes
No	Yes	No	No	No
No	Yes	No	No	No
Yes	Yes	Yes	Yes	Yes
No	Yes	No	No	No
No	Yes	No	No	No
No	Yes	No	No	No

No	Yes	No	No	No
No	Yes	No	No	No
Yes	Yes	No	No	No
No	Yes	No	No	No
Yes	Yes	Yes	Yes	Yes
No	No	No	No	No
No	No	No	No	No
No	No	No	No	No
No	No	No	No	No
No	Yes	Yes	Yes	No
Yes	Yes	No	No	No
Yes	Yes	No	No	No
No	No	No	Yes	No
Yes	Yes	No	No	No
Yes	Yes	No	No	No
No	No	No	No	No
No	No	No	Yes	No
No	No	Yes	No	No
No	No	No	No	No
Yes	No	No	No	No
Yes	Yes	No	No	No
Yes	Yes	Yes	Yes	Yes
Yes	Yes	No	No	No
Yes	Yes	Yes	Yes	Yes
No	No	No	Yes	No
Yes	No	No	No	No
Yes	Yes	Yes	Yes	Yes
Yes	Yes	Yes	Yes	Yes
Yes	Yes	No	No	No
Yes	No	No	No	No
No	Yes	No	Yes	No
No	Yes	No	Yes	No
No	Yes	No	No	No
Yes	Yes	Yes	Yes	Yes
Yes	Yes	No	No	No
No	No	Yes	No	No
No	No	No	No	No
No	No	No	Yes	No
Yes	Yes	Yes	Yes	Yes
No	No	No	Yes	No
No	No	No	Yes	No
Yes	Yes	No	No	No
No	No	No	Yes	No
Yes	Yes	No	No	No
Yes	Yes	Yes	Yes	No
Yes	Yes	Yes	No	Yes
No	Yes	No	No	No
Yes	Yes	Yes	Yes	No
Yes	Yes	Yes	Yes	Yes
Yes	Yes	No	No	No
Yes	No	No	No	No
Yes	Yes	Yes	Yes	No
Yes	Yes	No	Yes	Yes
No	No	No	Yes	No

No	No	No	Yes	No
No	No	No	No	No
No	No	No	Yes	No
No	No	No	Yes	No
No	No	No	Yes	No
No	Yes	No	No	No
Yes	Yes	Yes	Yes	Yes
No	No	No	No	No
No	No	No	No	No
Yes	Yes	Yes	Yes	No
No	No	No	Yes	No
Yes	Yes	No	Yes	No
Yes	Yes	No	Yes	No
No	Yes	No	Yes	No
No	No	No	No	No
Yes	Yes	Yes	Yes	Yes
Yes	Yes	No	No	No
Yes	Yes	No	No	No
No	No	No	No	No
Yes	Yes	Yes	Yes	No
Yes	Yes	No	Yes	No
Yes	Yes	Yes	Yes	No
No	No	No	Yes	No
No	No	No	Yes	No
No	No	No	Yes	No
No	No	No	Yes	No
Yes	Yes	No	No	No
Yes	Yes	No	No	No
No	Yes	No	Yes	No
Yes	Yes	Yes	No	No
No	No	No	No	No
Yes	Yes	No	No	No
No	No	No	No	No
Yes	Yes	No	Yes	No
Yes	Yes	No	No	No
No	No	No	Yes	No
No	No	No	No	No
No	No	No	Yes	No
Yes	Yes	Yes	No	No
No	No	Yes	No	No
Yes	Yes	Yes	No	No
Yes	Yes	No	Yes	No
Yes	Yes	Yes	Yes	Yes
No	Yes	No	No	No
No	No	No	Yes	No
No	No	No	No	No
No	No	No	Yes	No
No	No	No	Yes	No
No	No	No	Yes	No
Yes	Yes	No	No	No
Yes	Yes	Yes	Yes	No
No	No	Yes	No	No
No	No	No	No	No
No	No	No	No	No

No	No	No	No	No
No	No	No	No	No
No	No	No	Yes	No
No	No	No	Yes	No
No	No	No	Yes	No
Yes	Yes	No	No	No
No	No	No	Yes	No
Yes	Yes	Yes	Yes	No
No	No	No	Yes	No
Yes	Yes	Yes	Yes	Yes
No	No	No	Yes	No
Yes	Yes	Yes	Yes	No
No	No	No	Yes	No
Yes	Yes	No	Yes	No
Yes	Yes	Yes	Yes	Yes
Yes	Yes	No	No	No
Yes	Yes	No	Yes	No
No	No	No	Yes	No
No	No	No	Yes	No
No	No	No	No	No
No	No	No	No	No
Yes	Yes	No	Yes	No
No	Yes	No	Yes	No
No	Yes	No	Yes	No
Yes	Yes	No	No	Yes
No	No	No	No	No
No	No	No	No	No
No	No	No	Yes	No
No	No	No	Yes	No
No	No	No	No	No
No	No	No	No	No
No	No	No	Yes	No
Yes	No	No	No	No
No	No	No	No	No
Yes	Yes	Yes	Yes	Yes
Yes	Yes	Yes	Yes	No
Yes	Yes	No	Yes	Yes
No	No	No	Yes	No
No	No	No	Yes	No
No	Yes	No	Yes	No
No	No	Yes	No	No
Yes	Yes	Yes	Yes	Yes
No	Yes	No	Yes	No
No	Yes	No	No	No
Yes	Yes	No	No	No
No	No	No	Yes	No
Yes	No	No	No	No
No	Yes	No	No	No
No	Yes	No	No	No
Yes	Yes	Yes	No	No
Yes	Yes	Yes	Yes	Yes
Yes	Yes	No	No	No
No	No	No	No	No
No	No	No	Yes	No

Yes	Yes	Yes	Yes	No
Yes	Yes	Yes	Yes	No
No	No	No	No	No
No	No	No	No	No
Yes	Yes	Yes	Yes	Yes
Yes	Yes	No	No	No
No	Yes	No	Yes	No
Yes	Yes	No	No	No
No	No	No	No	No
No	No	No	No	No
Yes	Yes	Yes	Yes	No
Yes	Yes	Yes	No	No
Yes	Yes	No	No	No
Yes	Yes	No	No	No
No	No	No	Yes	No
No	No	No	No	No
No	No	No	No	No
No	No	No	Yes	No
No	No	No	Yes	No
No	No	No	No	No
No	Yes	No	No	No
Yes	Yes	No	No	No
No	No	No	No	No
No	No	No	Yes	No
No	No	No	Yes	No
No	No	Yes	Yes	No
No	No	No	Yes	No
No	No	No	No	No
No	No	No	No	No
No	No	No	Yes	No
No	No	No	Yes	No
No	No	Yes	Yes	No
Yes	No	No	No	No
Yes	Yes	No	Yes	No

SANGER CGC(05/30/2017)
Yes
Yes
Yes
Yes
Yes
No
No
No
Yes
No
Yes
No
Yes
Yes
Yes
Yes
No
No
No
Yes
Yes
No
No
Yes
No
Yes
No
Yes
No
No
Yes
Yes
No
No
Yes
No
Yes
No
Yes
Yes
Yes
No
No
No
No
No
No
No
Yes
No
Yes
Yes
No

Yes
No
Yes
Yes
Yes
Yes
No
No
Yes
Yes
No
No
No
No
Yes
Yes
No
Yes
No
No
Yes
Yes
Yes
No
Yes
Yes
Yes
Yes
No
No
No
Yes
Yes
Yes
Yes
Yes
Yes
Yes
Yes
Yes
Yes
Yes
Yes
Yes
Yes
Yes
Yes
Yes
No
Yes
No
Yes
No
Yes

Yes
Yes
No
Yes
Yes
Yes
Yes
No
Yes
Yes
Yes
Yes
Yes
Yes
Yes
Yes
Yes
No
Yes
No
Yes
Yes
Yes
Yes
No
No
Yes
No
No
No
No
No
Yes
Yes
Yes
No
Yes
Yes
Yes
Yes
Yes
Yes
No
No
Yes
Yes
No
Yes
Yes
Yes
No
Yes
No
Yes

No
Yes
No
No
Yes
Yes
No
Yes
No
Yes
Yes
No
Yes
Yes
Yes
Yes
Yes
No
No
Yes
Yes
Yes
Yes
No
Yes
Yes
Yes
No
No
No
Yes
Yes
No
No
Yes
No
No
No
Yes
Yes
No
Yes
No
No
No
Yes
Yes
No
Yes
Yes
No
Yes
Yes
Yes

No
No
Yes
Yes
Yes
Yes
No
No
No
No
No
Yes
Yes
No
Yes
No
No
Yes
No
No
No
No
No
No
Yes
No
No
No
Yes
No
No
Yes
Yes
No
No
Yes
Yes
Yes
No
No
No
Yes
No
Yes
No
Yes
No
No
No
No
No
No
No
Yes

Yes
Yes
Yes
Yes
Yes
Yes
Yes
Yes
No
Yes
No
No
Yes
No
Yes
No
Yes
Yes
Yes
Yes
Yes
No
Yes
Yes
No
Yes
Yes
No
No
Yes
No
Yes
Yes
Yes
Yes
Yes
Yes
No
Yes
Yes
Yes
Yes
No
Yes
Yes
Yes
Yes
Yes
No
No
No
No
No
No

No
No
Yes
Yes
Yes
Yes
Yes
Yes
Yes
Yes
Yes
Yes
No
Yes
Yes
No
No
Yes
No
Yes
Yes
Yes
Yes
Yes
No
Yes
Yes
No
Yes
No
No
No
No
No
Yes
Yes
Yes
Yes
No
No
No
No
No
Yes
No
No
Yes
Yes
No
Yes
Yes
Yes
Yes
No

No
Yes
No
No
No
No
Yes
No
Yes
No
No
No
No
Yes
Yes
No
Yes
Yes
No
No
No
No
No
No
No
No
No
No
No
No
No
No
No
No
Yes
No
No
No
No
No
No
No
No
Yes
No
No
No
No
Yes
No
No
Yes
Yes
Yes

Yes
Yes
Yes
Yes
Yes
No
Yes
No
Yes
Yes
Yes
Yes
Yes
No
Yes
Yes
No
No
No
Yes
Yes
No
No
No
No
Yes
Yes
Yes
Yes
No
Yes
No
No
No
Yes
Yes
No
Yes
Yes
No
No
No
No
No
No
No
No
No
Yes
No
No
No
Yes
Yes

Yes
Yes
Yes
No
No
Yes
Yes
Yes
No
Yes
No
No
Yes
Yes
Yes
Yes
Yes
Yes
No
Yes
Yes
No
Yes
No
Yes
No
Yes
Yes
No
Yes
Yes
No
No
Yes
No
Yes
Yes
Yes
Yes
Yes
No
Yes
Yes
Yes
Yes
Yes
Yes
Yes
Yes
No
No
No
No
No

Yes
No
Yes
No
Yes
Yes
No
Yes
Yes
Yes
Yes
Yes
Yes
Yes
No
No
No
Yes
No
No
Yes
No
No
No
Yes
Yes
No
Yes
Yes
No
No
Yes
No
Yes
No
No
No
Yes
No
No
Yes
Yes
Yes
Yes
Yes
Yes
Yes
Yes
No
No
No
Yes
No
Yes

Yes
No
Yes
No
Yes
Yes
No
No
No
Yes
Yes
Yes
Yes
Yes
Yes
Yes
Yes
Yes
Yes
Yes
No
Yes
Yes
Yes
No
No
No
Yes
Yes
Yes
No
Yes
Yes
Yes
No
Yes
No
Yes
Yes
Yes
No
Yes
Yes
Yes
No
Yes
Yes
Yes
No
No
Yes
Yes
Yes
No

No
Yes
Yes
Yes
Yes
Yes
Yes
No
Yes
No
Yes
No
Yes
Yes
No
Yes
Yes
Yes
Yes
Yes
No
Yes
Yes
No
No
No
No
Yes
No
No
No
No
No
Yes
Yes
Yes
Yes
Yes
Yes
No
No
No
Yes
No
No
No
Yes
Yes
Yes
Yes
Yes
No
No
No

Yes
No
No
No
Yes
Yes
Yes
No
No
No
No
Yes
Yes
No
No
Yes
No
No
Yes
Yes
Yes
No
No
No
Yes
No
Yes
No
Yes
Yes
Yes
Yes
Yes
Yes
Yes
Yes
Yes
No
Yes
No
No
Yes
Yes
Yes
No
Yes
Yes
Yes
Yes
Yes
No
No
No
Yes

No
No
Yes
Yes
Yes
Yes
No
No
No
Yes
Yes
No
No
Yes
Yes
No
Yes
No
No
Yes
Yes
No
Yes
Yes
No
Yes
No
No
Yes
No
No
No
No
Yes
No
Yes
Yes
Yes
No
No
Yes
Yes
Yes
No
Yes
Yes
No
No
Yes
No
No
Yes
Yes
No

No
Yes
Yes
No
Yes
No
Yes
Yes
Yes
Yes
Yes
No
No
No
No
No
No
Yes
Yes
No
Yes
Yes
No
No
No
No
Yes
No
Yes
No
Yes
Yes
Yes
Yes
Yes
Yes
No
No
No
No
No
No
No
No
No
No
Yes
Yes
No
No
Yes
No
No
No

No
No
No
No
Yes
Yes
No
No
Yes
No
Yes
No
Yes
No
No
Yes
No
Yes
Yes
No
No
Yes
Yes
Yes
No
No
Yes
Yes
Yes
Yes
No
No
No
Yes
No
No
Yes
No
Yes
No
No
No
No
No
Yes
No
No
Yes
Yes
No
No
Yes
Yes
Yes

Yes
Yes
Yes
Yes
Yes
No
Yes
No
No
Yes
No
No
Yes
Yes
Yes
Yes
No
No
Yes
Yes
Yes
Yes
No
Yes
Yes
Yes
No
No
Yes
Yes
Yes
No
Yes
Yes
Yes
Yes
No
No
No
No
Yes
Yes
Yes
No
Yes
Yes
Yes
No
No
No
Yes
No
No
No

No
No
No
Yes
Yes
Yes
No
Yes
No
Yes
No
Yes
Yes
Yes
Yes
No
Yes
Yes
Yes
Yes
Yes
No
No
No
Yes
Yes
Yes
No
Yes
Yes
Yes
Yes
No
Yes
Yes
Yes
Yes
No
No
No
No
Yes
No
Yes
No
Yes
Yes
No
No
No
Yes
No
Yes
No

Yes
Yes
Yes
Yes
Yes
Yes
No
No
Yes
Yes
Yes
No
No
No
No
Yes
No
No
Yes
No
No
Yes
No
Yes
No
No
No
Yes
Yes
Yes
Yes
No
No
Yes
