# Supplementary material for: Inflation of tumor mutation burden by tumor-only sequencing in under-represented groups
Source: NPJ Precis Oncol. 2021 Mar 19;5:22. doi: 10.1038/s41698-021-00164-5 (PMC7979755; doi:10.1038/s41698-021-00164-5)
Supplement: Supplementary file 2 — Data [file 41698_2021_164_MOESM2_ESM.pdf]

See "EXAMPLE" tab which gives an example of a c

**README: Complete one row for each dataset supporting the figures, tables and results reported in your manuscript**
**Datasets supporting your figures**
Data supporting Figure 1
Data supporting Figure 2
**Datasets supporting your tables**
Data supporting Table 1
**Datasets included in your supplementary information files**
Data supporting supplementary file 2
**Other datasets generated as part of this study**
You may find it useful to check the subheadings of your Results and Methods s
(add rows as necessary)

completed summary form

Data file name(s)	**Data file format & file type** (of data *supporting* Figures, etc; not of Figures themselves)	**Location of data file(s) or repository name**
Supporting_Data, worksheets "Fig 1a+2" and "Fig 1b"	excel	Institutional file storage
Supporting_Data, worksheet "Fig 1a+2"	excel	Institutional file storage
Supporting_Data, worksheet "Fig 1a+2"	excel	Institutional file storage
Supporting_Data, worksheets "Supplementary Figure 2" , "Supplementary Figure 3-a", "Supplementary Figure 3-b	excel	Institutional file storage
ections as these often align with data underlying your manuscript.		

**If the dataset is in a repository, enter the accession number, DOI or link to the dataset**

**How the data file will be accessible by other researchers after your manuscript has been published**
Available from npj Precision Oncology
Available from npj Precision Oncology
Available from npj Precision Oncology
Available from npj Precision Oncology

If applicable, please explain why the dataset will not be publicly available (note: *npj Precision Oncology* strongly encourages data sharing)

**Would you like to publish these files for free in the journal repository? Yes/No** https://springernature.figshare.com/npjprecisiononcology
Yes
Yes
Yes
Yes
